# 3D Multi-Ion Corrosion Model in Hierarchically Structured Cementitious Materials Obtained from Nano-XCT Data

**DOI:** 10.3390/ma16145094

**Published:** 2023-07-19

**Authors:** Krzysztof Szyszkiewicz-Warzecha, Jakub Stec, Jan Deja, Artur Łagosz, Anna Górska, Kristina Kutukova, Ehrenfried Zschech, Robert Filipek

**Affiliations:** 1 Faculty of Materials Science and Ceramics, AGH-University of Krakow, al. Mickiewicza 30, 30-059 Krakow, Poland; 2 deepXscan GmbH, Zeppelinstr. 1, 01324 Dresden, Germany; 3 Research Area Nanomaterials, Brandenburg University of Technology Cottbus-Senftenberg, Platz der Deutschen Einheit 1, 03046 Cottbus, Germany

**Keywords:** 3D corrosion model, real 3D concrete microstructure, nano-XCT based geometry, corrosion of reinforcement, multi-ion transport, hierarchical concrete structure

## Abstract

Corrosion of steel reinforcements in concrete constructions is a worldwide problem. To assess the degradation of rebars in reinforced concrete, an accurate description of electric current, potential and concentrations of various species present in the concrete matrix is necessary. Although the concrete matrix is a heterogeneous porous material with intricate microstructure, mass transport has been treated in a homogeneous material so far, modifying bulk transport coefficients by additional factors (porosity, constrictivity, tortuosity), which led to so-called effective coefficients (e.g., diffusivity). This study presents an approach where the real 3D microstructure of concrete is obtained from high-resolution X-ray computed tomography (XCT), processed to generate a mesh for finite element method (FEM) computations, and finally combined with a multi-species system of transport and electric potential equations. This methodology allows for a more realistic description of ion movements and reactions in the bulk concrete and on the rebar surface and, consequently, a better evaluation of anodic and cathodic currents, ultimately responsible for the loss of reinforcement mass and its location. The results of this study are compared with a state-of-the-art model and numerical calculations for 2D and 3D geometries.

## 1. Introduction

The corrosion of steel bars has received much attention over the last decades. The survey of literature presented below is supposed to pay the reader’s attention to the following aspects: (i) description of cementitious materials at various scales; (ii) nano X-ray tomography studies of cementitious materials; and (iii) modeling of ion transport in cementitious materials.

### 1.1. Description of Cementitious Materials at Various Scales

Accurate models describing the properties of concrete, a highly complex structural material, must cover all length scales from macro to nano, including the smallest length scale relevant to concrete properties and material transport processes, which is the scale of electron interactions with atoms and ions. However, concrete properties (physicochemical, structural, functional) ultimately manifest at larger lengths and time scales. Thus nanoscale models must be linked to models on meso- and macroscopic scales, a well-known paradigm called the “bottom-up” approach [1]. Future advances in concrete materials need the design and control of their tailored properties and manufacturing processes using several advanced simulation tools, each suitable from electronic through atomic, molecular, nanoscale, microscale up to macroscale description.

The C-S-H binding phase, a reaction product between anhydrous calcium silicates and water, comprises small nanoparticles of around 5 nm average diameter that form a gel-like network of variable stoichiometry [2,3]. One of the first models of the molecular structure of C-S-H obtained by numerical calculations was reported by Pellenq et al. [4]. The results were consistent with experimental data on average composition, density, scattering and spectroscopic signatures [5]. Such models can create a database of atomic configurations, corresponding defect attributes, and mechanical properties for various C-S-H chemical compositions. These models provide guidelines to predict an optimum composition concerning mechanical properties and overall performance [4].

At macro-level simulations of concrete and, more generally—cementitious materials—the continuum model-based computations using mass and charge transport conservation laws at the macro-scale have been extensively employed with great success [6]. The primary numerical method used for solving models at this level is the finite element method (FEM). However, the finite difference method (FDM) is occasionally employed, especially if the model can be reduced to 1D.

The next modelling level is the mesoscale level, in which aggregates and cement paste are differentiated. They can also include, optionally, interfacial transition zones (ITZ) [7]. Finite element method, multigrid methods (especially for computation of large heterogeneous mesoscale models), and lattice models are the most appropriate. However, their success crucially depends on accurate modeling of the mesoscale geometry. Hence the topology of the porous material is usually either constructed either from X-ray computed tomography (XCT) (image-based modelling) or by in silico meso-structure generation (parametric modelling) [8].

Microscale models correspond to the level where the cement paste is described in terms of its constituents and where chemistry and thermodynamics begin to play a role [5]. It is the critical scale for studying and understanding the fracture behavior of cement paste [9]. Two aspects at this level of modelling should be underlined: the first is how to obtain a realistic microstructure, and the second is how to include the local micromechanical properties of different phases in the cement paste [10]. The first issue can be tackled by numerical models or experiments. A high-resolution microstructure of the material can be obtained using backscattered electron imaging (BSE) in the scanning electron microscope (SEM) [11]. However, the main shortcoming of this technique is that it provides only 2D information.

In contrast, micro-XCT provides nondestructive 3D information on the interior of cementitious materials. It has been successfully applied in the study of cement-based materials since the end of the 20th century [12]. It has a typical spatial resolution in the micrometre range, sometimes down to 1.0 µm or even 0.5 µm. As for the second issue, the micromechanical properties of constituting components of cementitious materials are usually derived from standard nano-indentation measurements [13]. Image-based models give accurate geometry of aggregates and voids, but computational efforts are high for the complex geometries obtained from 2D and 3D images. Attention should also be paid to the fact that imaged regions may not statistically represent the size and spatial distributions of aggregates in the sample studied.

The next level is the sub-micro level, where the colloidal and gel-like properties C-S-H are analyzed and built into the models. This level explicitly assumes the existence of C-S-H particles and studies their aggregations [5].

Finally, simulations and modelling of cement-based materials shrink to the nanoscale, where the atomistic description and quantum effects must be employed. Here fundamental methods like ab-initio, Monte Carlo, molecular dynamics (MD), or density functional theory (DFT) are applied [14,15,16]. Since atomic level simulations are well suited for the description of crystalline structures, most studies on this level have been performed to characterize the atomic structure of the cementitious near-crystalline mineral phases like calcium silicate hydrates (C-S-H) or similar phases, such as calcium aluminate silicate hydrates (C-A-S-H). For instance, Mohamad et al. [17] were able to asses 14 Å C-S-H (of tobermorite type) defect structures and their stability using DFT and MD calculations. They also developed a building-block description and notation to represent and encode the full atomic structure of C-S-H models in a simple, readable string of characters. Classical MD simulations with force field potential were used by Kumar et al. [18] to test the structural stability of various synthesized C-S-H structures with compositionally uniform calcium silicate hydrate phases and Ca/Si ratios in the range 1.0–2.0. Mishra et al. [19] carried out force field atomistic simulations to elucidate surface properties, its interactions with organic molecules, and agglomerations at the molecular scale for tricalcium silicate (C_3_S)—the predominant mineral phase in cement. These models are the first steps toward quantitative simulations of cementitious materials from the nanoscale, and further extensions to other phases are expected. 

### 1.2. Nano X-ray Tomography Studies of Cementitious Materials

Laboratory X-ray computed tomography (XCT) is essential for nondestructive 3D imaging of cementitious materials. The extensive review of XCT application for cement-based materials, from the methodical background to the various applications, was published by Brisard et al. [20]. While micro-XCT, with a spatial resolution down to 0.5 µm is a well-established technique, the potential of the lens-based nano-XCT, with a resolution in the sub-100 nm range, has been demonstrated at synchrotron radiation sources [21,22,23,24,25,26,27,28] (see Table 1). For example, synchrotron-radiation ptychography is used to illustrate the best possible spatial resolution in X-ray imaging, however, for the price of higher efforts in data analysis. For a more systematic study of the hierarchical porous structure of cementitious materials [29], laboratory nano-XCT studies are needed in addition to micro-XCT studies. These laboratory nano-XCT studies allow us to systematically investigate the effect of process parameters on the 3D morphology of cementitious materials. It is also possible to image kinetic processes in these materials that cause degradation and failure. 

Robuschi et al. [33] used a combination of neutron and X-ray tomography to observe corrosion products in reinforced concrete structures nondestructively. The particular interest was in the region adjacent to the reinforcement bar, where corrosion products are accommodated without causing additional stresses on the concrete matrix. In a compact review of relevant monitoring and detection apparatus for reinforced concrete structures, Li et al. [34] draw attention to the effective evaluation of the images and the fact that most of the images still need to be judged by experienced operators. They also underlined XCT as one of the analytical techniques for monitoring steel reinforcement corrosion in concrete. Wang et al. [35] proposed a method of monitoring the deformation in reinforced concrete under accelerated corrosion conditions based on a combination of XCT and digital image correlation (DIC), and they calculated the evolution of strain and stress fields. Zeng et al. [36] investigated water imbibition (spontaneous water uptakes in the capillary system) in ultra-thin concrete samples using (microfocus) X-ray radiography. Chung et al. [37] determined the pore size distribution of concrete with air-entraining admixtures using micro-XCT data applying both the volume-based method and the chord-length distribution in 3D for the calculation. Brisard et al. [20] reviewed both micro- and nano-XCT, including their broad application fields. The review underlines that none of the other prevalent techniques in concrete research allows the nondestructive visualization of the 3D microstructure and that XCT provides the spatial distribution of porosity, connectivity, and tortuosity of cementitious materials. Their analysis of more than 400 papers leads to the conclusion that many applications of micro-XCT to cementitious materials can now be considered routine tasks. Yu et al. [38] applied XCT to study a special type of concrete—the so-called pervious concrete, produced with little or no fine aggregate, resulting in a large, open pore structure. Based on XCT, the 2D/3D pore topology was analyzed. Apart from the standard characteristics of porous materials (porosity, tortuosity, and pores size), they calculated more indexes for describing the pore structure of pervious concrete and analyzed the effect of pore structure on permeability for the direct measurement of the moisture content in concrete based on XCT data, contrast agents, such as CsCl, are often introduced into the water to improve the image contrast. However, the use of such agents is undesirable because of their impact on water viscosity, capillary bond, or chemical reactions in the concrete matrix. Oesch et al. [39] addressed this problem and proposed an additional image calibration procedure to correct water-related X-ray scattering. Song et al. [40] calculated three parameters relevant to fluid transport (percolating porosity $\phi_{percol}$, critical pore diameter *d*_c_, and average tortuosity τ) from FIB/SEM image stacks and inferred permeability *K* by Katz–Thompson equation $K=d_{c}^{2}\phi_{percol}/(226\tau )$ for a high-performance concrete (HPC) using 3D focused ion beam/scanning electron microscopy (FIB/SEM). 

### 1.3. Modeling of Ions Transport in Cementitious Materials

Tian et al. [41] performed 3D numerical simulations for chloride transport using commercial software COMSOL ver. 5.6. The transport model was very basic. Only Fick’s diffusion of one species (no coupling to other ions and no electric potential) was considered. Still, the main focus was applying XCT to reconstruct concrete’s real 3D meso- and microstructure. To achieve this goal, an improved method—considering the X-ray signal attenuation in the specimen—was applied to extract the meso-structure of concrete. The grayscale image of concrete was divided into several annular subregions, and a self-adaptive grayscale threshold was selected to perform the segment of each annular region before the binarization processing. Rao and Pan [42] analyzed two methods of electrochemical prevention of rebar corrosion: electrical injection of corrosion inhibitors (EICI) and electrochemical removal of chlorides (ERC) based on diffusion, electro-migration, and hydraulic-migration mathematical models. To obtain the internal structure of the concrete as an input for their simulations, the authors used 3D-reconstructed XCT data.

Several cementitious materials were designed and exposed to chloride solutions in [43] to assess the influence of different parameters such as fly ash content, mass fraction, water/binder (W/B) ratio etc., on the chloride ions transport in unsaturated concrete. However, the mathematical transport model was simple, based on the Fick law for the diffusion of Cl^−^ and Darcy’s law for the migration velocity of water or moisture without directly accounting for other ions and 3D geometry. A relatively comprehensive model of chloride transport in saturated and unsaturated concrete has been recently advanced by Zhang et al. [44]. It is based on the known phenomenon of transport in cementitious materials, including chemical activity and membrane potential, effects of pore structure evolution on diffusivity, diffusivity dependence on concentration, chloride binding capacity, and microstructure change due to hydration reactions. This model is restricted to only one species; however, the innovative numerical solution method is implemented as a discrete conduit network generated from the mesoscale geometry of the Lattice Discrete Particle Modeling (LDPM). It allows to tailor the flow through the concrete internal structure. Chloride ingress into a mortar matrix has also been recently considered by Chen et al. [45], where a 2D model based on the classical Nernst–Planck flux (dilute electrolyte approximation) with a first-order chloride binding reaction was used to simulate the distribution of bound and free chloride ions. Tian et al. [46] analyzed the influence of the thickness of interfacial transition zone ITZ on the chloride ions transport in concrete using a numerical model at mesoscale in 1D and 2D geometries based on a one-component Fick’s law only to account for the distribution of chloride ions. The diffusivity of ITZ was parametrized by the volume fraction of aggregate and the volume fraction of ITZ. 

Dai et al. [47] developed a 3D mesoscale model as a three-phase composite material consisting of a mortar matrix, aggregate, and interfacial transition zones (ITZ) to simulate concrete cracking induced by corrosion product expansion of helical strands. In [48], authors analyzed the influences of various factors, such as the water/cement ratio, ITZ thickness, coarse aggregate volume fraction, and maximum coarse aggregate size in concrete, on the chloride diffusion in the ITZ. Kumar and Mukherjee [49] performed an extensive parametric study based on a simple Fick’s law 2D model of diffusion to obtain the numerical relationship between diffusion coefficient and various concrete parameters describing its micro-topology, such as coefficient of porosity and interconnection, coefficient of ITZ thickness and relative diffusivity, coefficient of aggregate ratio or coefficient of degree of aggregate size gradation.

The study of the cracking of a concrete cover due to the non-uniform corrosion of corner-located rebar was performed by Jin et al. [50]. Most works focused on the side-located rebar where chlorides penetrate from one direction, while in the case of the steel bar at the corner, chlorides can penetrate from both sides, making the corner-located rebar more susceptible to rust. A three-dimensional random aggregate model of concrete with concrete regarded as a three-phase composite composed of coarse aggregates, mortar matrix and the interfacial transition zones (ITZs) and the cracking of the concrete protective layer caused by the non-uniform corrosion of the corner-located rebar was simulated.

Filipek et al. [51] used a 3D continuum model with corrosion reactions on the rebar surface. They pointed out some possible difficulties and ambiguities that may arise while using standard tests, for example, promoted by RILEM or ASTM, for assessing steel corrosion in concrete by measuring the electric potential distribution over the concrete cover. Jin et al. [50] studied a nonlinear bond interaction between a ribbed steel bar and concrete to investigate the bond failure process of the ribbed steel/concrete interface. A 3D mesoscale finite element approach was used based on the plastic-damage continuum model for concrete, which assumes that the main failure mechanisms of the concrete are cracking in tension and crushing in compression. The interaction mechanism between ribbed steel bars and concrete was assumed to be friction resistance and mechanical interaction. Isgor and Weiss [52] used a standard multi-species reactive transport with volumetric water content convective mode to calculate the electrical resistivity, formation factor, chloride binding and chloride ingress as a tool for the prediction of reinforcing steel corrosion. They also included the effects of ionic activities in their model as a modified Davies equation for activity coefficient. Sun et al. [53] used a standard continuum model of ion transport based on the Nernst–Planck flux to predict a time-dependent distribution of sulfate ions in concrete. The model also considers the chemical reactions of sulfate ions to form gypsum and ettringite, composition-dependent diffusivities and the spatially dependent distribution of pore size in ITZ. Five components were considered: two mobile ions $\left(Ca^{2+},\;SO_{4}^{2-}\right)$ and three immobile neutral species: calcium aluminates, ettringite and gypsum. Computations were performed in 2D geometry.

Another approach to describe chloride ingress at the macro scale, often found in literature, is probabilistic modeling. The chloride transport, commonly described by the 2nd Fick law of diffusion, can be solved numerically or analytically. However, its solution is not affected by the material’s microstructure or other factors, such as the environment and resulting degradation processes [54]. Considering it within the stochastic framework, it is possible to investigate the influence of uncertainties associated with material properties, geometry of structure or environmental conditions. Probabilistic modeling has been applied in the analysis of chloride ingress in concrete structures with macrocracks [55], unsaturated concrete [56] or the analysis of pitting corrosion [57]. The literature survey shows that even up-to-date advanced studies of the transport and corrosion in reinforced concrete are characterized either by essentially homogeneous treatment of the concrete matrix (with possibly including several species, typically Na^+^, K^+^, Cl^−^, OH^−^, oxygen) and non-linear electrode kinetics (Butler–Volmer or Tafel equations) or by more sophisticated treatment of the concrete matrix (including its real micro-structures), but with a simple transport model (only oxygen). The goal of this paper is to fill this gap by a combination of the following two aspects in one computational model: (i) sub-micro scale X-ray computed tomography (XCT); and (ii) a multi-ion reaction model for the description of the corrosion of steel in reinforced concrete.

## 2. Materials and Methods

### 2.1. Sample Preparation and Characterization 

The nano XCT studies were performed on mortar samples with weight proportions of sand (Kopalnia Surowców Mineralnych Dziergowice, Górażdże Heidelberg Cement Group, Dziergowice, Poland), cement CEM I 42.5R (Górażdże Cement SA, Górażdże Heidelberg Cement Group, Chorula, Poland), and water equal 3:1:0.5. The value of the water-to-cement ratio, *w/c* = 0.5, considered the minimum component requirements for concretes working under the influence of chlorides. Mortar cubes measuring 150 mm, 100 mm, and mortar beams of 40 × 40 × 160 mm^3^ were cast in molds. The weighed ingredients were mixed in a mixer (HL120, Hobart GmbH, Offenburg, Germany) for 3 min, and immediately after mixing, the samples were formed on a vibrating table (B005, ToRoPol, Warsaw, Poland) for 15 s. 

The strengths of the prepared mortar samples were assessed after 2, 28, and 90 days of hardening, and the absorbency properties after 28 and 90 days of curing in water. The porosity measurements were performed using mercury intrusion porosimetry (MIP) by a PoreMaster 60 instrument (Quantachrome Instruments, Boynton Beach, FL, USA), covering the conventional pore diameter range of 3 × 10^−3^ to 2 × 10^2^ µm. The flexural and compressive strength evaluations were conducted on samples with dimensions outlined in EN 196-1 [58] and prepared in accordance with the procedure described in this standard. Absorbency was measured for 100 mm edge cubic samples. The specimens for porosity tests were obtained from fragments of 40 × 40 × 160 mm^3^ beams, which were not damaged during the direct measurement of the flexural strength. The results of these tests are summarized in Table 2. 

In the presented model and all calculations, the gel pore fraction of CEM I-based specimens was assessed from the literature review. Jennings [59] proposed a model for the CSH phase, in which the low-density (LD-CSH) and high-density (HD-CSH) can be distinguished. The gel porosities are 37.3% (LD-CSH) and 23.7% (HD-CSH), and they should be considered as intrinsic properties of them [60]. Vandamme [61] determined gel porosities for various *w/c* ratios (0.15–0.40) based on the nanoindentation investigations. Muller [62,63] measured gel porosity using ^1^H nuclear magnetic resonance (NMR) for other *w/c* ratios. In this paper, we investigate samples with a *w/c* ratio of 0.50. Thus, we assumed a gel porosity of 30.3%.

### 2.2. Nano-XCT Study

**Figure 1:**
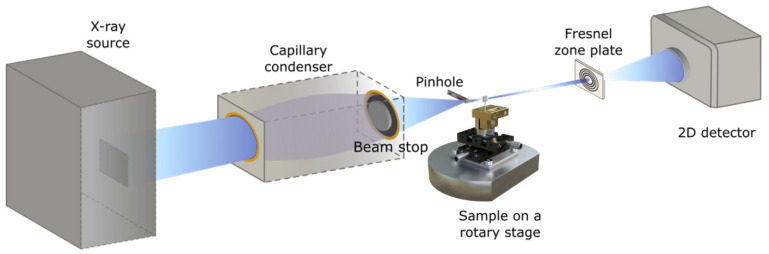
Scheme of the nano-XCT experiment.

Laboratory X-ray microscopy data of a cement sample were obtained using an Xradia nXCT-100 instrument (Xradia, Pleasanton, CA, USA)—Figure 1. This transmission X-ray microscope (TXM) consists of a rotating anode as an X-ray source (Cu-Kα radiation, 8 keV photon energy), a capillary condenser optic, a Fresnel zone plate (FZP) as an objective, and a scintillator/CCD detector system. In the full-field imaging mode, the field of view (FOV) is 65 µm × 65 µm, resulting in a pixel size of 65 nm for camera binning 1 (1024 × 1024 pixels). This setup provides an almost parallel-beam geometry. Therefore a 180° rotation is sufficient for (XCT). A tomography tilt series of 601 images was recorded within an exposure time per image of 120 s. 3D tomographic reconstruction is performed with the standard Xradia software package XMReconstructor 9.0.

The sample was prepared by chipping off a smaller piece of 40 to 80 µm to meet the transmission requirement of the cement-based material for 8 keV and to fit the FOV. Manipulation with the sample as preparing, selecting, mounting, and marking with a gold bead (1.5–3 µm size, Alfa Aesar) was performed under an optical microscope. The sample was fixed on the holder with the Gorilla super glue (Gorilla Glue, Inc., Cincinnati, OH, USA). 

**Figure 2:**
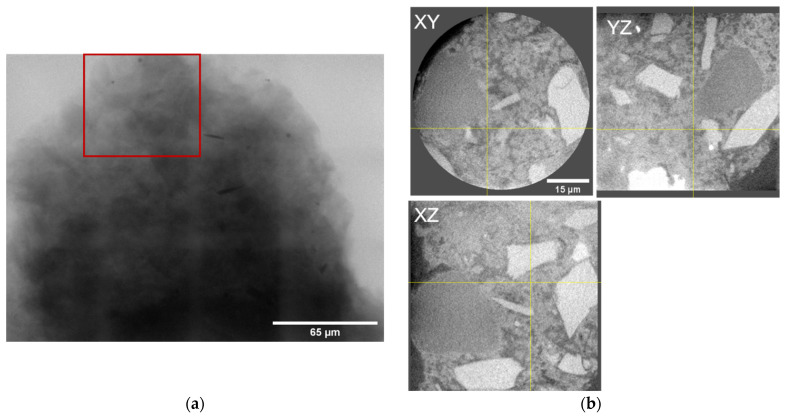
(**a**) Observed zone of the sample—Region of Interest (ROI)—red box, (**b**) Example of the virtual cross-sections in all three dimensions—XY, YZ and XZ.

The size of the observed volume was 65 × 65 × 65 µm^3^. In Figure 2b, one can distinguish four components: cement material, capillary pores, and two filler grains: multiple brighter grains and one large dark grain.

### 2.3. XCT Data Processing and 3D/2D Mesh Generation

**Figure 3:**
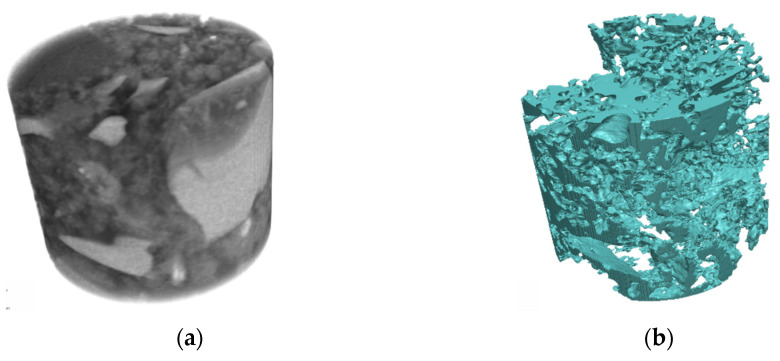

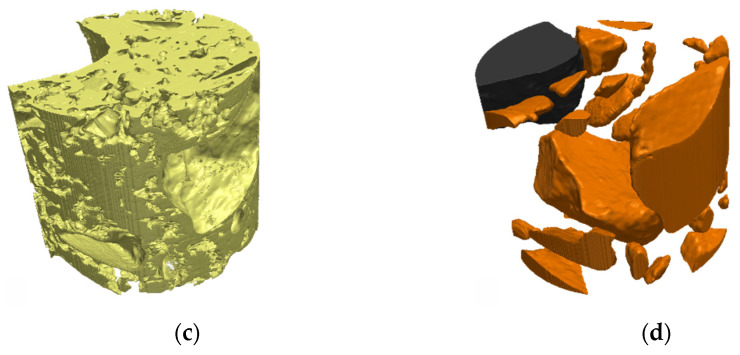
(**a**) Unsegmented XCT data and results of phase segmentation: (**b**) capillary pores, (**c**) cement with gel pores and (**d**) filler grains.

The first step of data processing, brightness, and contrast adjustments was done using the ImageJ ver. 1.53t [64] software. From the initial XCT data, a smaller cylinder (ø = 46.8 µm, *h* = 39 µm) was cut. The cropped data was imported into the Simpleware ScanIP ver. U-2022.12 [65] software. Using the appropriate threshold, four masks representing: cement with gel pores (yellow), capillary pores (blue), light grains (orange) and dark grains (black)—Figure 3. Segmented data was then used to create 2D and 3D meshes for numerical simulation using the FE module implemented in Simpleware ScanIP ver. U-2022.12. The meshes were created using the FE model type, with the export type dedicated to COMSOL Multiphysics software (Nastran file format). The mesh creation algorithm was selected as +FE Free, and all elements were tetrahedral (linear). The parameters chosen for 3D mesh were target minimum length (1.4 µm), target maximum error (0.23 µm), maximum edge length (26 µm), target number of elements across layer (0.5), surface change rate (100) and volume change rate (90). Those parameters allowed us to obtain the 3D mesh made of 2,221,768 elements.

**Figure 4:**
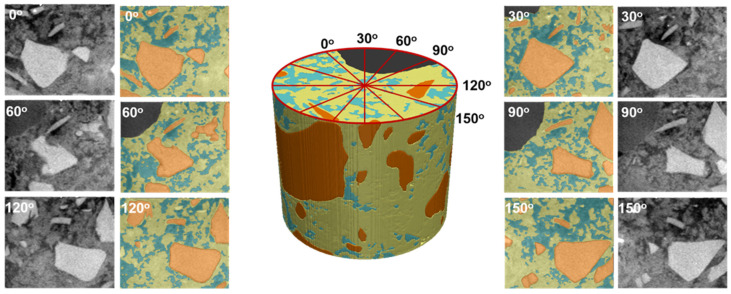
A 3D geometry with marked selected cross-sections is used to create 2D geometries for numerical simulations before and after segmentation: cement paste (yellow), capillary pores (blue), and grains (orange and dark grey).

To create the 2D meshes for numerical simulations, the 3D data was reduced from 400 to 4 layers in the X direction. To receive 2D mesh, the target number of elements across layers was 0.01, while the rest parameters were set the same as for 3D mesh. To analyze different 2D cross-sections, the 3D data was rotated by 30°, 60°, 90°, 120° and 150°, and then was cropped and used to create 2D meshes. Consequently, we created a set of six 2D meshes—see Figure 4.

## 3. 3D Multi-Ion Corrosion Model in Cementitious Materials

### 3.1. Multi-Component Diffusion-Migration Transport in Porous Media

We consider *N* ionic and *N*_0_ non-ionic (neutral) species, respectively, which can move in a 3D bounded region in space $\Omega \subset {\mathbb{R}}^{3}$ with porosity coefficient 0<ϕ≤1. Their concentrations (mol m^−3^), which depend on position ***x*** ∈ Ω and time *t* ≥ 0, will be denoted as $c_{1}(x,t),\ldots ,c_{N}(x,t),$ $c_{N+1}(x,t),\ldots ,c_{N+N_{0}}(x,t),$ and the corresponding molar fluxes as $J_{1},\ldots ,J_{N},$ $J_{N+1},\ldots ,J_{N+N_{0}}$ (mol m^−2^ s^−1^).

The movements of all components in Ω are primarily constrained by the mass conservation law of each species *i*, which in a continuum model has the following form of partial differential equations.

(1) $$\phi \frac{\partial c_{i}}{\partial t}\;+\;\mathrm{div}\;J_{i}=R_{i},\;i=1,\ldots ,N+N_{0}.$$

The Nernst–Planck flux expression for the *i*-th species

(2) $$J_{i}=-D_{i}^{eff}\nabla c_{i}+B_{i}^{eff}c_{i}E.$$

splits the driving force for the motion of species into two parts: the Fickian diffusion $\left(-D_{i}^{eff}\nabla c_{i}\right)$ term and electric field induced migration term $(B_{i}^{eff}c_{i}E).$ Here, **E** is the electric field $(V\;m^{-1}),$ $D_{i}^{eff}$ is the effective diffusion coefficient (m s^−1^) and $B_{i}^{eff}$ is the effective mobility coefficient (m^2^ V^−1^ s^−1^). In a typical application of the flux (2), the Nernst–Einstein relation between diffusion and mobility coefficients is assumed.

(3) $$B_{i}=\frac{z_{i}e}{k_{B}T}D_{i}=\frac{z_{i}F}{RT}D_{i}.$$

To calculate the electric field **E** (or the electric potential *φ*) in the bulk of the macroscopic region Ω we have to add one more equation to the system of Equations (1) and (2). Charge conservation law

(4) $$\frac{\partial \rho }{\partial t}+\mathrm{div}\;j=0$$

where $j=F\sum_{i-1}^{n}z_{i}J_{i}$ is the electric current density (A m^−2^), or the Poisson equation for the electric potential.

(5) $$\mathrm{div}(\varepsilon_{0}\varepsilon_{r}\nabla \varphi )=\rho ,$$

where the volume charge density $\rho =F\sum_{i=1}^{n}z_{i}c_{i}$ (C m^−3^) is necessary. In the domains on the scale of centimeters or higher, the deviation from the electroneutrality condition is small and Equation (5) can be substituted by Equation (6)

(6) $$\sum_{i=1}^{N}z_{i}c_{i}(x,t)=0,\;(x\in \Omega ),$$

which yields div j=0.

For obtaining a unique solution, the boundary conditions must supplement the above equations describing physical behavior on the system boundary ∂Ω. For charge transfer redox reactions, the flux conditions are of the following type:

(7) $$-n\cdot J_{i}=g_{i}(x,\varphi ,c_{1},\ldots ,c_{N+N_{0}})\;\mathrm{on}\;\mathrm{boundary}\;\partial \Omega$$

$i=1,\ldots ,N+N_{0}.$ Conditions (7) postulate the normal component of the mass flux through the boundary depending on the local environment properties (position, concentration of species, electric field or potential). The general form expressed in (7) allows for a uniform description of different situations because the functions *g_i_* depends on the position ***x*** ∈ ∂Ω. It means that in some places, it may define, for an instant, a redox reaction. In other—an oxidation reaction or blocking behavior for a particular species. For example, if the reaction of iron dissolution $\mathrm{Fe}\to {\mathrm{Fe}}^{2+}+2e^{-}$ takes place and we assume that it is governed by the Butler–Volmer kinetics equation, then the relevant boundary condition reads:

(8) $$-n\cdot J_{{\mathrm{Fe}}^{2+}}=\frac{i_{\mathrm{Fe}/{\mathrm{Fe}}^{2+}}^{0}}{2F}\exp\left(\frac{\alpha_{a}F}{RT}(\varphi_{\mathrm{Fe}}-\varphi -E_{\mathrm{Fe}/{\mathrm{Fe}}^{2+}}^{eq})\right)\;\mathrm{on}\;\partial \Omega_{\mathrm{Fe}}.$$

If on some part Γ ⊆ ∂Ω of the boundary, an anodic current density $i_{loc}=const$ is imposed by a galvanostat, and then the boundary condition would be:

(9) $$-n\cdot \left(\sum_{i=1}^{N}z_{i}J_{i}\right)=\frac{i_{loc}}{F}\;\mathrm{on}\;\Gamma .$$

### 3.2. A 3D Model of Current and Potential Distribution in Reinforced Concrete; Five Species Na^+^, OH^−^, Fe^2+^, Cl^−^, O_2_ and Potential 

We present an extension of the model [66] for obtaining current, mass, and potential distribution in the context of chloride-induced corrosion of steel bars in reinforced concrete. The main objective of the cited work was to show how one can compute mass transfer in a corroding rebar-concrete system which in turn could be used to assess a reduction of the cross-section area of reinforcement and to predict the increase of the volume of the corrosion products. However, the authors based their simulations on a simplified model in which only a transport of oxygen coupled with anodic dissolution of iron and cathodic reduction of oxygen by the Tafel-type kinetics were considered. The electric potential was obtained by the Laplace equation with no ionic species present. This form of description is known in electrochemistry as the primary current and potential distribution [66].

**Figure 5:**
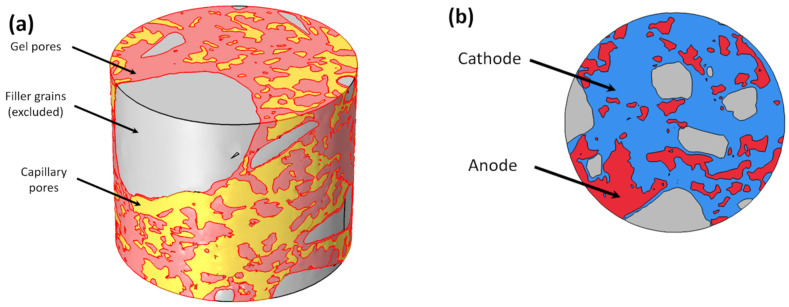
(**a**) Domains for computations and (**b**) cathodic/anodic sites for boundary conditions at the bottom of the cylinder.

To get a more realistic model, we consider processes of multi-component transport which take place inside the concrete and reactions on the re-bar surface embedded in the concrete matrix. Five components are considered: four ions Na^+^, Cl^−^, OH^−^, Fe^2+^ and oxygen, O_2_. Moreover, the whole physical space occupied by a concrete sample is divided into three regions (Figure 5a) obtained from the XCT scanning (Figure 3): capillary pores, cement with gel pores, and filler grains. The filler grains domain is excluded from computations. Capillary pores and cement with gel pores are treated separately and seamed together by the flux continuity at the interface between these two domains. Equations in capillary pores treat this domain as a liquid electrolyte solution, but equations in the gel pores domain treat it as a porous medium (with porosity coefficient).

In the corrosion model, charge transfer (redox) reactions on the surface of a rebar (14) and bulk (homogeneous) reactions in electrolytes are considered:

(10) $${\mathrm{Fe}}^{2+}+2{\mathrm{OH}}^{-}\;\to \;{\mathrm{Fe}(\mathrm{OH})}_{2}$$

(11) $$4\mathrm{Fe}{\left(\mathrm{OH}\right)}_{2}+\;O_{2}+2H_{2}O\to 4\mathrm{Fe}{\left(\mathrm{OH}\right)}_{3}$$

In this paper, we consider only one homogeneous reaction (10). Reactions (10) and (11) will be the subject of a separate paper. Using the Nernst–Planck flux, Fick’s flux, mass balance law, and electroneutrality condition instead of the Poisson equation for electric potential, we have the following set of partial differential-algebraic equations (PDAEs):

(12) $$\left\{\begin{array}{l} \frac{\partial c_{i}}{\partial t}\;+\;\nabla \cdot J_{i}=R_{i},\;J_{i}=-D_{i}\nabla c_{i}-z_{i}\frac{\;F}{RT}D_{i}c_{i}\nabla \varphi ,\;\\ \sum_{i=1}^{4}z_{i}c_{i}=0, \end{array}\right.$$

where $i={\mathrm{Na}}^{+},\;{\mathrm{OH}}^{-},\;{\mathrm{Fe}}^{2+},\;{\mathrm{Cl}}^{-},\;O_{2}$ with charge numbers, $z_{{\mathrm{Na}}^{+}}=+1,\;$$z_{{\mathrm{OH}}^{-}}=-1,\;$$z_{{\mathrm{Fe}}^{2+}}=+2,\;$$z_{{\mathrm{Cl}}^{-}}=-1,$ *F* is the Faraday constant, *R* is the universal gas constant, and *T* is the absolute temperature.

Reaction terms are dictated by the only homogeneous reaction (10):

(13) $$R_{{\mathrm{Na}}^{+}}=0,\;R_{{\mathrm{OH}}^{-}}=-2kc_{{\mathrm{Fe}}^{2+}}c_{{\mathrm{OH}}^{-}},\;R_{{\mathrm{Fe}}^{2+}}=-kc_{{\mathrm{Fe}}^{2+}}c_{{\mathrm{OH}}^{-}},\;R_{{\mathrm{Cl}}^{-}}=0,$$

which will be expressed as $R_{{\mathrm{OH}}^{-}}=-2R,\;R_{{\mathrm{Fe}}^{2+}}=-R$ with $R=k\;c_{{\mathrm{Fe}}^{2+}}c_{{\mathrm{OH}}^{-}},$ where *k* is the overall homogeneous rate constant.

**Boundary conditions (for mass balance).** The corrosion reactions that we consider are

(14) $$\begin{array}{l} \mathrm{iron}\;\mathrm{oxidation}:\;\mathrm{Fe}\;\to \;{\mathrm{Fe}}^{2+}+2e^{-}\\ \mathrm{oxygen}\;\mathrm{reduction}:\;O_{2}+2H_{2}O+4e^{-}\to 4{\mathrm{OH}}^{-} \end{array}$$

The Butler–Volmer equation, which connects the charge transfer overpotential with the rate of reaction on the surface, serves as the boundary condition for species ${\mathrm{Fe}}^{2+},\;O_{2},$ and ${\mathrm{OH}}^{-}.$ Hence, we have the following conditions (Figure 5b):

(15) $$\begin{array}{l} \mathrm{on}\;\mathrm{anode}:\;-n\cdot J_{{\mathrm{Fe}}^{2+}}=\frac{1}{2F}i_{\mathrm{Fe}},\\ \mathrm{on}\;\mathrm{cathode}:\;-n\cdot J_{O_{2}}=\frac{1}{4F}i_{O_{2}},\;-n\cdot J_{{\mathrm{OH}}^{-}}\;=\;\frac{1}{F}i_{O_{2}},\\ i_{\mathrm{Fe}}=i_{\mathrm{Fe}}^{0}\cdot \exp\left(\alpha_{\mathrm{Fe}}\frac{F}{RT}(U_{app}-\varphi -E_{\mathrm{Fe}}^{eq})\right),\;\\ i_{O_{2}}=-i_{O_{2}}^{0}\cdot (c_{O_{2}}/c_{ref})\cdot \exp\left(-\alpha_{O_{2}}\frac{F}{RT}(0-\varphi -E_{O_{2}}^{eq})\right), \end{array}$$

where we applied a common electrochemical convention that an anodic current is positive and a cathodic current is negative. The boundary conditions at the cathode satisfy a balance equation

(16) $${\left.(n\cdot J_{{\mathrm{OH}}^{-}})\;\right|}_{\mathrm{cathode}}\;+\;4\;{\left.\left(n\cdot J_{O_{2}}\right)\right|}_{\mathrm{cathode}}=0,$$

which is a consequence of the redox reaction (14).

The rest boundary conditions assume blocking walls which means the zero-flux conditions

(17) $$-n\cdot J_{i}=0,\;(i={\mathrm{Na}}^{+},\;{\mathrm{OH}}^{-},\;{\mathrm{Fe}}^{2+},\;{\mathrm{Cl}}^{-},\;O_{2}).$$

The differential-algebraic system (12) is not easy to solve it in this form, so to tackle the problem, we transform it by symmetrical embedding the electroneutrality condition. We will not present here the full derivation but only the final equations (details are provided in Appendix A), which take the form of conservation laws with artificial flux that corresponds to the equation of electric potential: 

Equations turn into:

(18) $$\begin{array}{l} \left(z_{{\mathrm{OH}}^{-}}-z_{{\mathrm{Na}}^{+}}\frac{D_{{\mathrm{Na}}^{+}}}{D_{{\mathrm{OH}}^{-}}}\right)\frac{\partial c_{{\mathrm{OH}}^{-}}}{\partial t}+\left(z_{{\mathrm{Fe}}^{2+}}-z_{{\mathrm{Na}}^{+}}\frac{D_{{\mathrm{Na}}^{+}}}{D_{{\mathrm{Fe}}^{+}}}\right)\frac{\partial c_{{\mathrm{Fe}}^{2+}}}{\partial t}+\left(z_{{\mathrm{Cl}}^{-}}-z_{{\mathrm{Na}}^{+}}\frac{D_{{\mathrm{Na}}^{+}}}{D_{{\mathrm{Cl}}^{-}}}\right)\frac{\partial c_{{\mathrm{Cl}}^{-}}}{\partial t}+\nabla \cdot J_{{\mathrm{Na}}^{+},\varphi }=R_{{\mathrm{Na}}^{+},\varphi }\\ \frac{\partial c_{{\mathrm{OH}}^{-}}}{\partial t}+\nabla \cdot J_{{\mathrm{OH}}^{-}}=R_{{\mathrm{OH}}^{-}},\;\frac{\partial c_{{\mathrm{Fe}}^{2+}}}{\partial t}+\nabla \cdot J_{{\mathrm{Fe}}^{2+}}=R_{{\mathrm{Fe}}^{2+}},\;\frac{\partial c_{{\mathrm{Cl}}^{-}}}{\partial t}+\nabla \cdot J_{{\mathrm{Cl}}^{-}}=0,\;\frac{\partial c_{O_{2}}}{\partial t}+\nabla \cdot J_{O_{2}}=0, \end{array}$$

where

(19) $$J_{{\mathrm{Na}}^{+},\varphi }=-(z_{{\mathrm{OH}}^{-}}-z_{{\mathrm{Na}}^{+}})D_{{\mathrm{Na}}^{+}}\nabla c_{{\mathrm{OH}}^{-}}-(z_{{\mathrm{Fe}}^{2+}}-z_{{\mathrm{Na}}^{+}})D_{{\mathrm{Na}}^{+}}\nabla c_{{\mathrm{Fe}}^{2+}}-(z_{{\mathrm{Cl}}^{-}}-z_{{\mathrm{Na}}^{+}})D_{{\mathrm{Cl}}^{-}}\nabla c_{{\mathrm{Cl}}^{-}}.$$

Reaction terms turn into:

(20) $$R_{{\mathrm{Na}}^{+},\varphi }=-z_{{\mathrm{Na}}^{+}}\left(2\frac{D_{{\mathrm{Na}}^{+}}}{D_{{\mathrm{OH}}^{-}}}+\frac{D_{{\mathrm{Na}}^{+}}}{D_{{\mathrm{Fe}}^{2+}}}\right)R,\;R_{{\mathrm{Fe}}^{2+}}=R,\;R_{{\mathrm{OH}}^{-}}=2R,\;R_{{\mathrm{Cl}}^{-}}=0,\;R_{O_{2}}=0,$$

where $R=-k\;c_{{\mathrm{Fe}}^{2+}}c_{{\mathrm{OH}}^{-}}.$

Boundary conditions turn into:

(21) $$\begin{array}{l} \mathrm{anode}:\;\\ -n\cdot J_{{\mathrm{Na}}^{+},\varphi }\;=\;-z_{{\mathrm{Na}}^{+}}\frac{D_{{\mathrm{Na}}^{+}}}{D_{{\mathrm{Fe}}^{2+}}}\frac{i_{\mathrm{Fe}}}{2F},\;-n\cdot J_{{\mathrm{Fe}}^{2+}}=\;\frac{i_{\mathrm{Fe}}}{2F},\;-n\cdot J_{{\mathrm{OH}}^{-}}=-n\cdot J_{{\mathrm{Cl}}^{-}}=-n\cdot J_{O_{2}}=0,\\ \mathrm{cathode}:\;\\ -n\cdot J_{{\mathrm{Na}}^{+},\varphi }=z_{{\mathrm{Na}}^{+}}\frac{D_{{\mathrm{Na}}^{+}}}{D_{{\mathrm{OH}}^{-}}}\frac{i_{O_{2}}}{F},\;-n\cdot J_{{\mathrm{OH}}^{-}}\;=-\;\frac{i_{O_{2}}}{F},\;-n\cdot J_{O_{2}}=\frac{i_{O_{2}}}{4F},\;-n\cdot J_{{\mathrm{Cl}}^{-}}=-n\cdot J_{{\mathrm{Fe}}^{2+}}=0. \end{array}$$

On these parts o, boundaries are electrically isolated or not penetrable.

(22) $$-n\cdot J_{i}=0,\;(i=\varphi ,\;{\mathrm{OH}}^{-},\;{\mathrm{Fe}}^{2+},\;{\mathrm{Cl}}^{-},\;O_{2}).$$

## 4. Results

The numerical study aimed to investigate the influence of the microstructure of cementitious material the on corrosion of the steel reinforcement. Calculations were performed using the 3D multi-ion corrosion model with the values of $i_{O_{2}},\;i_{\mathrm{Fe}},\;\beta_{O_{2}},\;\beta_{\mathrm{Fe}}$ and $D_{O_{2}}$ taken from [66]. Notice that diffusion coefficients are reduced by three orders of magnitude compared with molecular (in pure water) coefficients to account for the porous nature of the concrete [67]. All parameters are summarized in Table 3.

**Figure 6:**
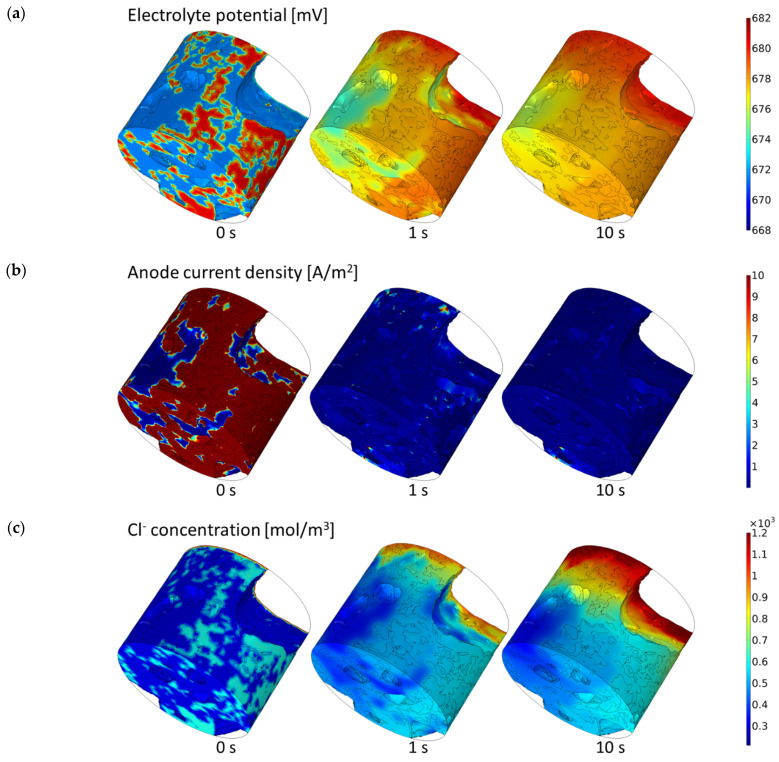

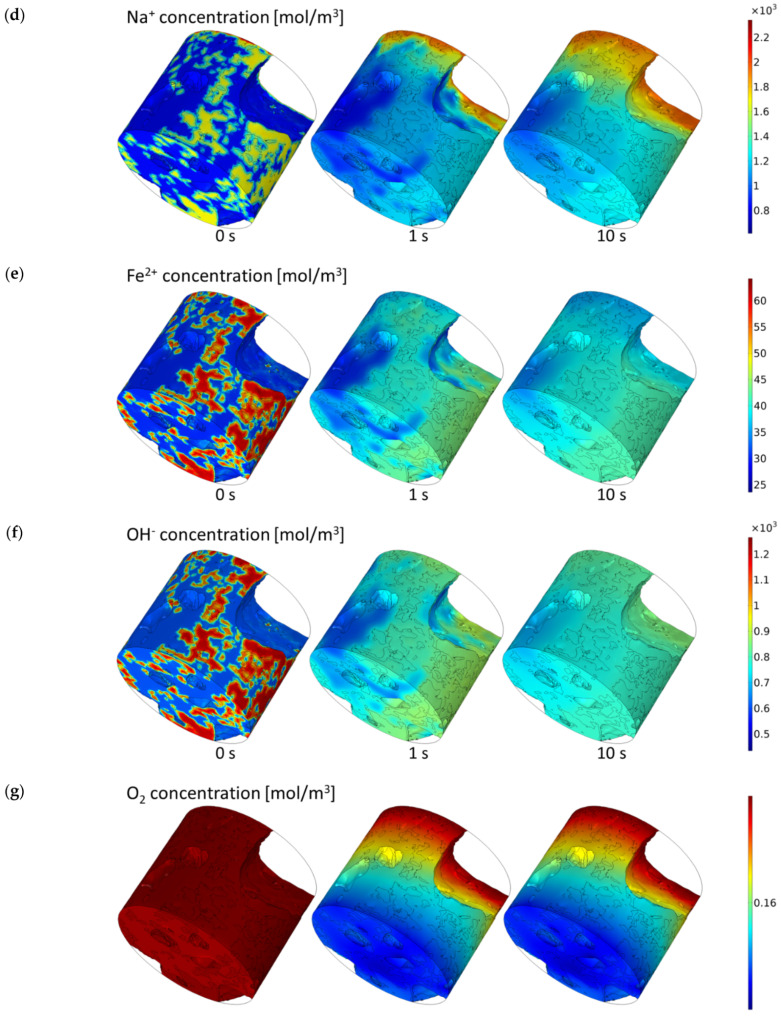
3D distribution of electric potential (**a**), anode current density (**b**), and concentration of species (**b**–**g**), respectively, in real cementitious microstructure for selected times.

**Figure 7:**
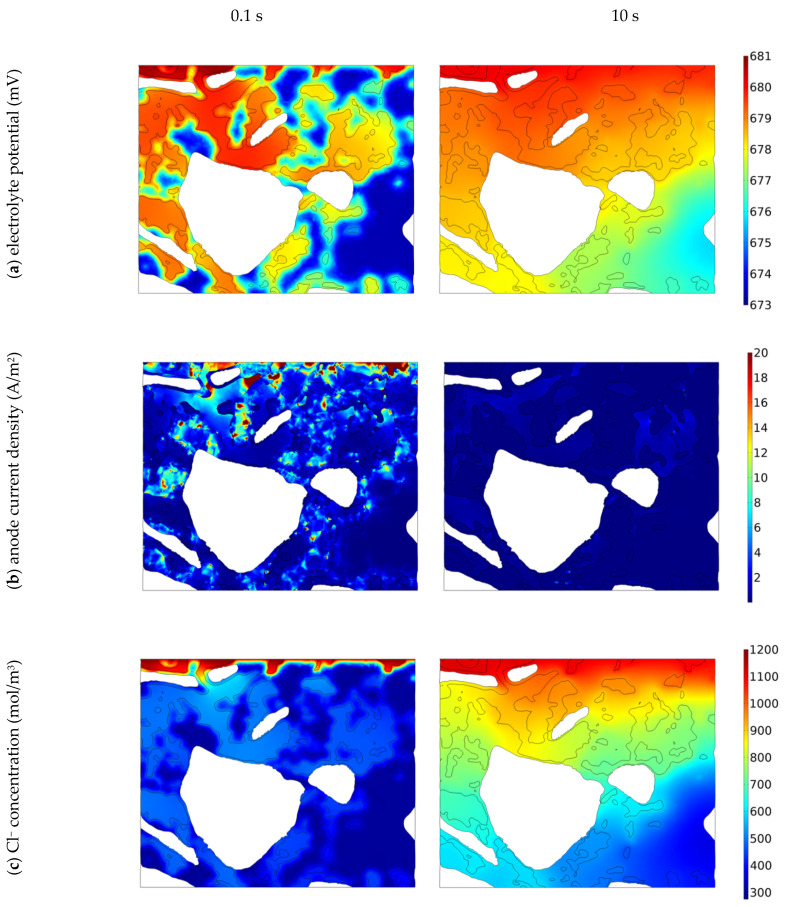

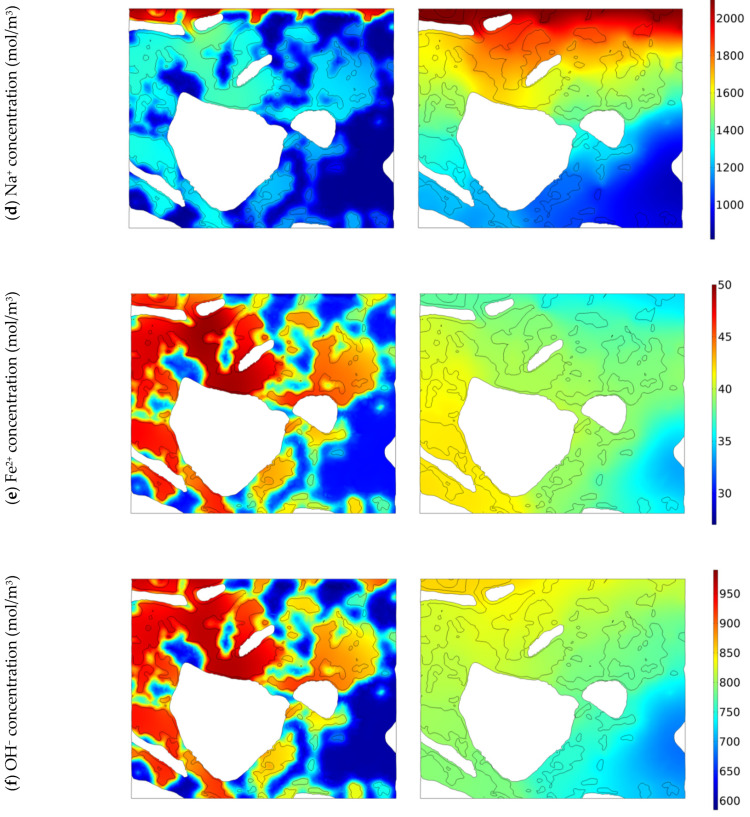

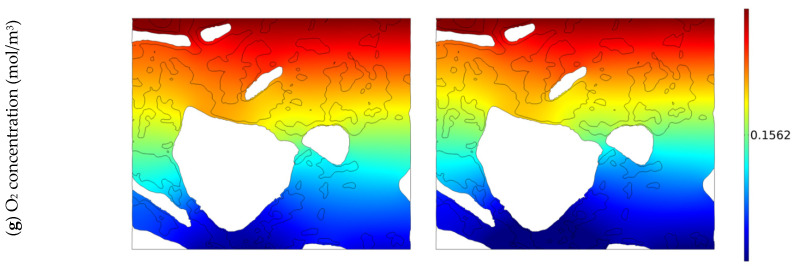
Electric potential (**a**), anode current density (**b**), and concentrations of species (**c**–**g**) at a selected cross-section of the sample (0 deg—see Figure 4) in real cementitious microstructure at two times: 0.1 and 10 s.

**Figure 8:**
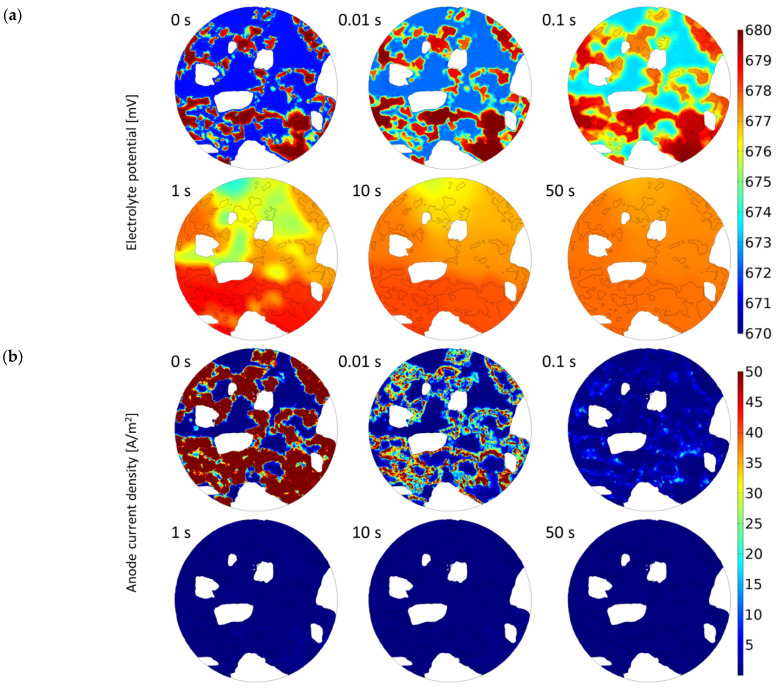

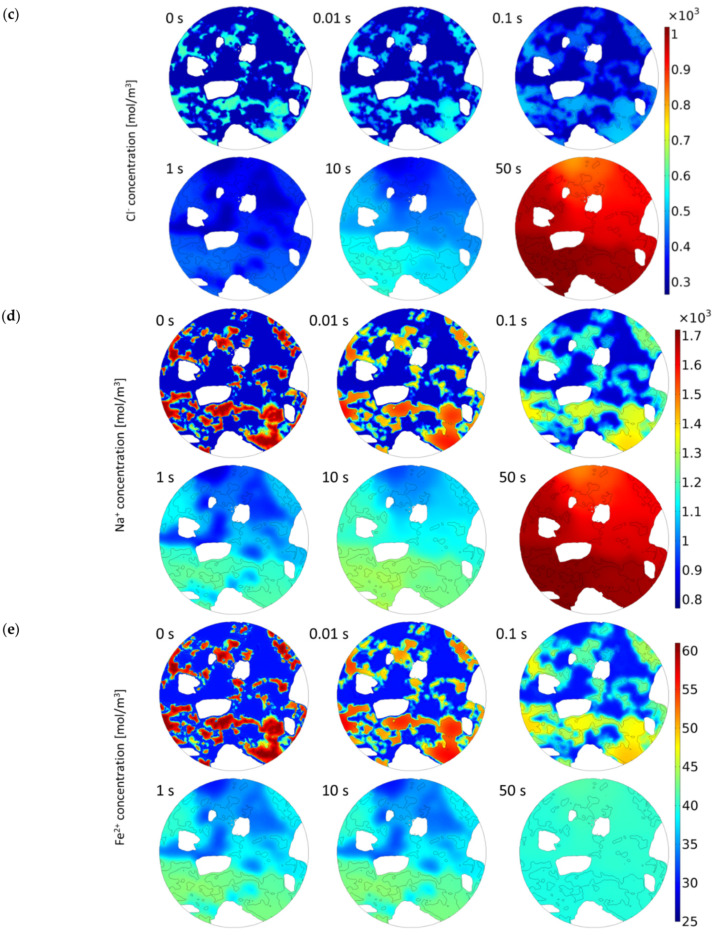

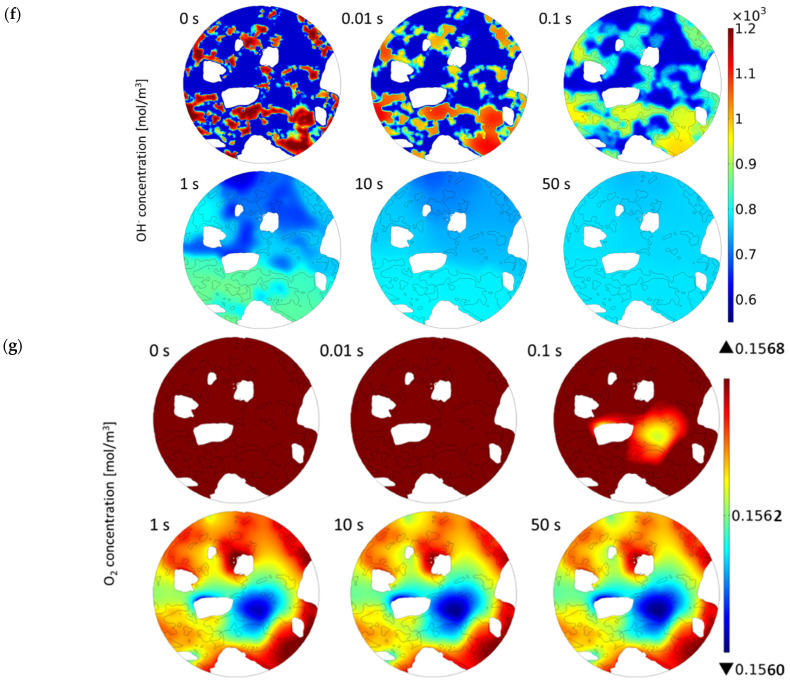
Electric potential (**a**), anode current density (**b**) and concentrations of species (**c**–**g**) near the reinforcement surface in real cementitious microstructure for selected times.

In this section, we will demonstrate the influence of a material’s microstructure on the distribution of electrical potential, current density, ions and oxygen concentration, directly and indirectly affecting the corrosion process. Figure 6 presents calculated electrical potential, anode current density, Na^+^, Cl^–^, OH^–^, Fe^2+^ ions and oxygen distribution in 3D cementitious material nano/microstructure for selected times: 0, 1 and 10 s. To visualize better the influence of a real microstructure of the cementitious material on the solution, the electric potential, current density, and concentrations of species at the selected cross-section of the sample (0 degrees) in the direction perpendicular to the top of the sample (see Figure 4) for selected times: 0.1 and 10 s are shown in Figure 7. Figure 8 shows solutions for selected times (0, 0.01, 0.1, 1 10, and 50 s) in the vicinity of the anode and cathode.

Figure 6 demonstrates that the sample’s microstructure containing capillary pores and mortar with gel porosity strongly influences the species concentrations and, consequently, the electrical potential distribution. As expected, the potential is higher near the anodic sites, generating a current density from anodic to cathodic zones. The potential distribution in the sample flattens with time, shifting towards more positive values. This flattening is related to more ions entering the sample, increasing the overall conductivity—Figure 14.

Both chloride and sodium ions steadily ingress into the sample from the top (contact with NaCl(*aq*) solution) and are bound by strong electrostatic interaction (near electroneutrality constraint). Hence their movement is strongly correlated (Figure 6b,c). But 10 s period is insufficient to move most of the salt to the rebar region.

Ferrous and hydroxide ions are produced on the rebar surface due to the oxidation and reduction reactions, respectively. Since the rate is not large, mainly evening out their concentrations is observed (which starts from some residual initial value (For better visualization of bulk homogenous reaction (10) some residual initial Fe^2+^ ions concentration which is different in capillary pores and the cement is assumed—Figure 8)). Moreover, the homogeneous reaction between Fe^2+^ and OH^−^ uses up these ions in bulk which can be seen in the overall decrease in their quantity in the sample (Figure 15).

The initial concentration of oxygen inside and outside the sample is the same. Hence there is no oxygen concentration gradient on the top of the sample at *t* = 0, and later oxygen practically does not enter the sample. But on the rebar, the cathodic reduction of oxygen leads to its consumption and a pronounced decrease in concentration over time (Figure 6f).

Some of the above processes can be visualized by considering 2D cross-section plots in Figure 8. For example, the initial concentration of oxygen near the rebar surface (0.16 mol/m^3^) with time since the rate of its consumption by oxygen reduction is much higher than its replenishment by diffusion transport. Similarly, Figure 8d shows that the initial high concentration of Fe^2+^ ions (red spots) drops even though there is a production of Fe^2+^ by anodic dissolution. However, the deposition reaction in the sample consumes Fe^2+^ ions quicker, giving the overall decrease in Fe^2+^ quantity.

## 5. Comparison of 3D Multi-Ion Corrosion Model with Ožbolt’s Model

**Figure 9:**
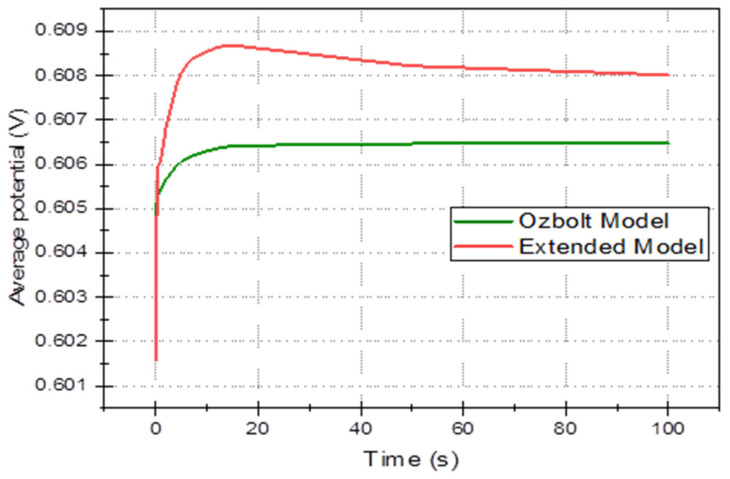
Average electric potential over the whole sample as a function of time for Ožbolt’s model and the extended model.

**Figure 10:**
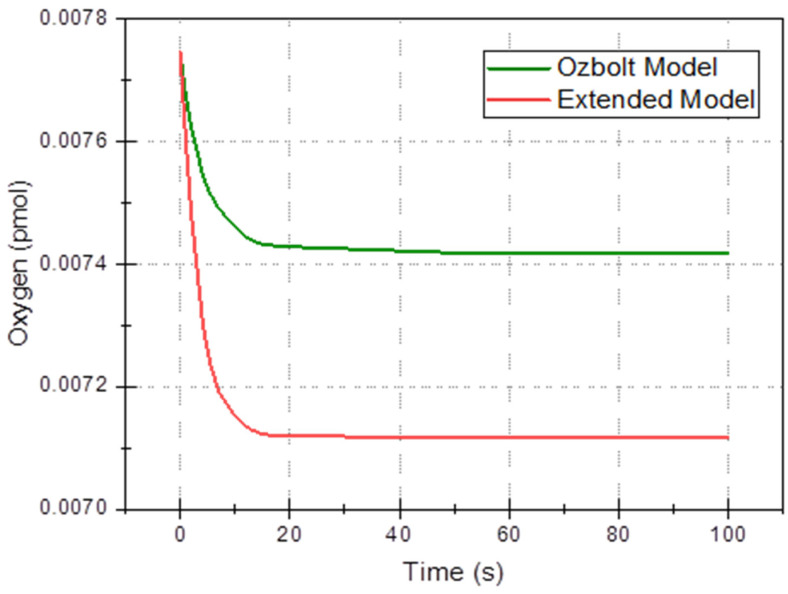
The total oxygen content in the sample is a function of time for Ožbolt’s and extended models.

**Figure 11:**
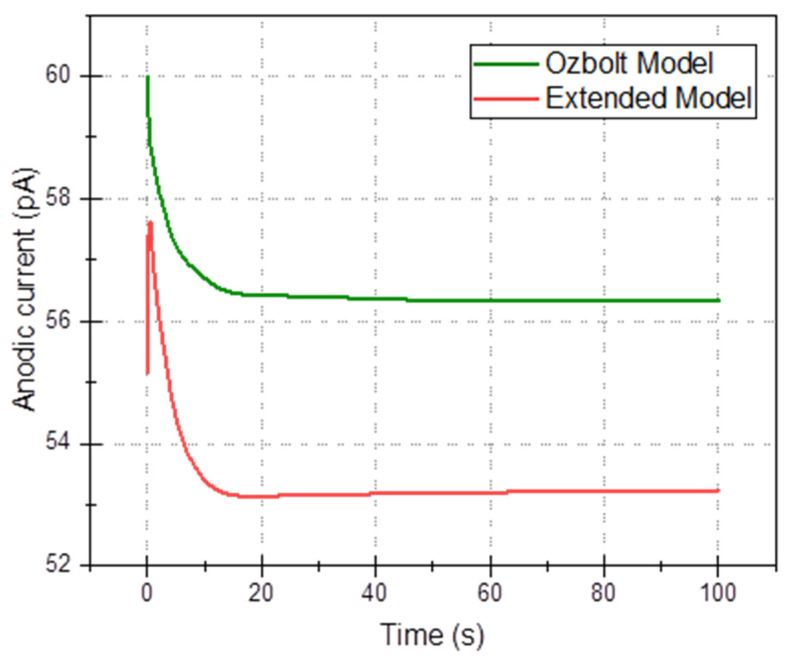
Comparison of total anodic current as a function of time for Ožbolt’s model and the extended model.

**Figure 12:**
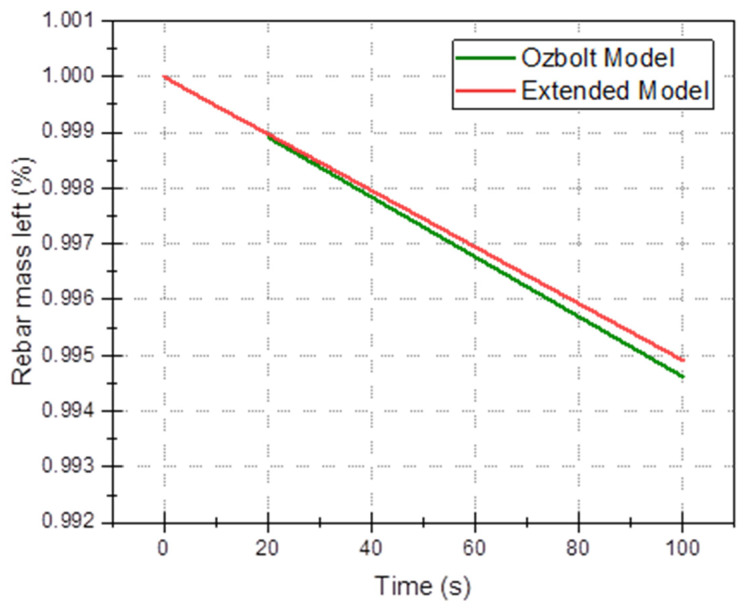
A drop of rebar mass due to anodic loss of iron (% of initial mass) for Ožbolt’s and extended models.

In this section, the 3D multi-ion corrosion model of rebars—see Section 3 (called further “the extended model”) is compared with a state-of-the-art model, in particular Ožbolt’s model [66] in the time domain. In the latter model, only oxygen and electric potential are used (current density on the electrode and in concrete are derived quantities), so for comparison, we selected four average quantities as functions of time: average electric potential, *φ*_avg_(*t*), (Figure 9), total oxygen content (Figure 10), total anodic current (Figure 11), and mass loss of rebar (due to anodic zones iron oxidation), (Figure 12). All common parameters, boundaries and initial conditions are the same in both simulations.

Figure 9 is shown that the electric potential settles at a steady value after some transitory interval in both models. However, the extended model predicts non-monotonic behavior, and the steady values differ. The electric potential in simplified models is expected to be monotone as potential is computed from the Laplace equation (Δφ = 0), which is time-independent, and the only slight dependence on time may come from boundary conditions, which in this case is only via cathodic reduction (in (15) equation for *i*_O2_) where the concentration of oxygen on the rebar surface can change. In the case of the extended model, however, the influence on the electric potential of several ions in the system is considered, resulting in a peak of potential. This behavior is also related to the characteristic dip of average conductivity (cf. Figure 14) at an early stage. Both these phenomena can be explained by the fact that initial values of ionic concentrations give average conductivity of around 1 S/m, but when the process starts, ferrous and hydroxide ions are consumed by the homogeneous reaction, which exceeds the rate of production at rebar leading to the general depletion of ions.

Our model also predicts a larger decrease in the oxygen concentration (Figure 10) which can be readily explained by the fact that it accounts for the consumption of OH^–^ ions, thus promoting cathodic reaction (oxygen reduction) (14), which uses up oxygen.

The above results and discussion show differences between simplified modelling based only on the oxygen diffusion and constant conductivity and more comprehensive modelling, which considers all species (oxygen and ions), possible homogeneous deposition reactions and non-constant electrical conductivity, are concluded. The difference in the predictions is demonstrated in Figure 9, Figure 10, Figure 11 and Figure 12. Of particular interest is Figure 12, where the loss of rebar mass is simulated over 100 s time for two models. From the visible trend, it is obvious that the difference increases with time.

## 6. The Influence of Selected Parameters on 3D Multi-Ion Corrosion Model Solutions

The 3D multi-ion corrosion model of reinforced concrete proposed in this paper is affected by various parameters. In this section, we investigate the influence of the rate of homogeneous reaction (10) and the oxygen diffusion coefficient on the dynamics of reinforcement corrosion.

### 6.1. The Influence of Homogeneous Rate Constant

**Figure 13:**
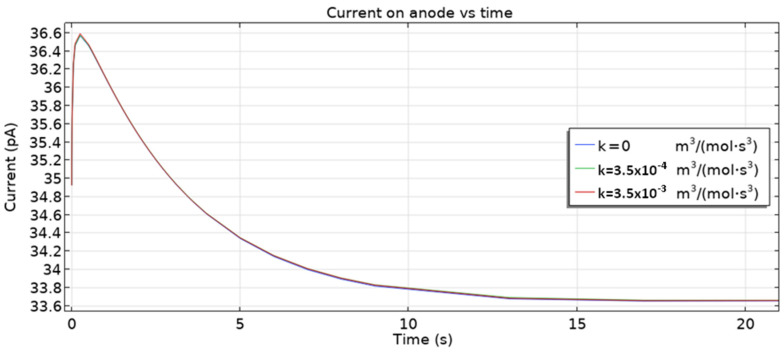
Current on the anode vs. time for two rates of homogeneous reaction and without the reaction (*k* = 0).

Figure 13 presents the dependence of the total anodic current on the rate of homogeneous reaction (10). The presence of the reaction has a negligible impact on the currents. It is caused by the fact that there is no Fe^2+^ concentration overpotential in the model. 

**Figure 14:**
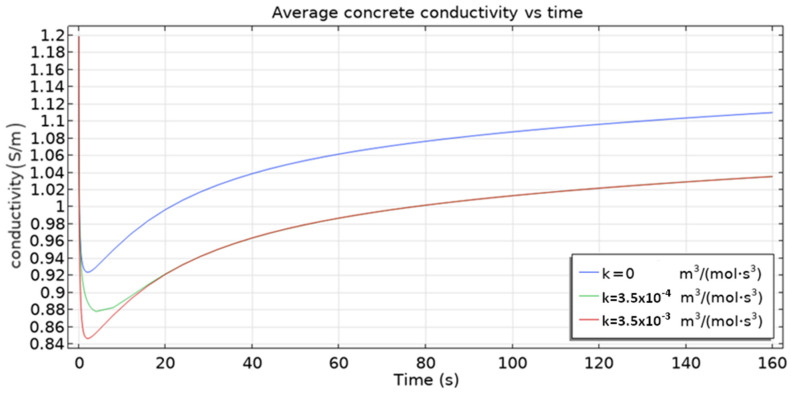
Time evolution of the average electric conductivity of the sample, σ (S/m) for selected rate constants *k*. With time more ions are present in the sample hence the increase of the overall conductivity after some transient period is observed.

The behavior of average conductivity (Figure 14) is particularly interesting. A sharp fall at the beginning followed by a slower increase until an almost steady state is reached after about 160 s is shown. The increase in the average conductivity is caused mainly by the increase in the total quantity of chloride ions that enter the sample.

**Figure 15:**
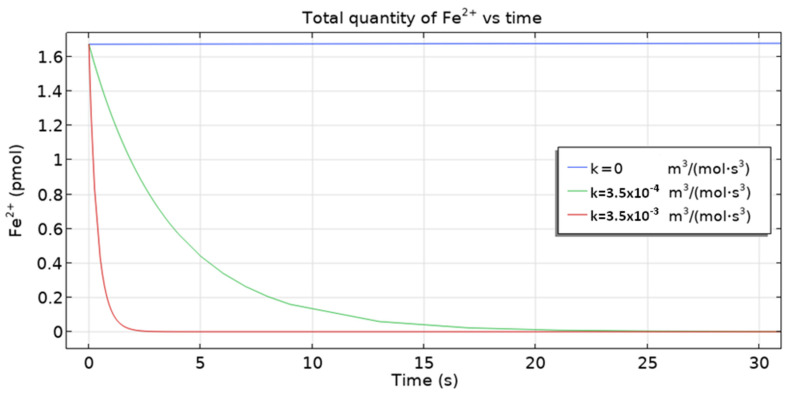
The total quantity of Fe^2+^ ion vs time for three homogeneous reaction rate constants.

**Figure 16:**
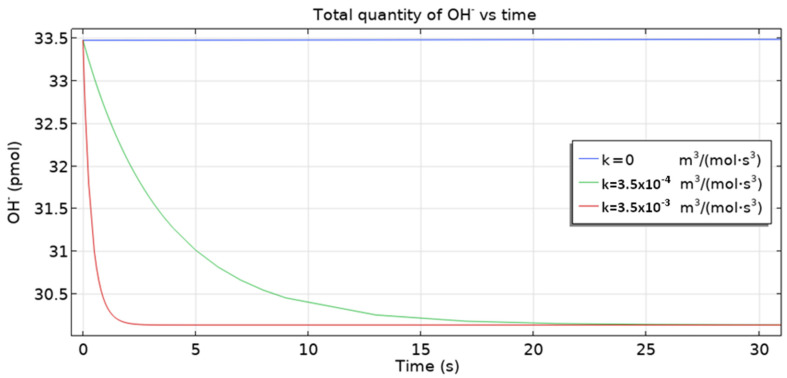
The total OH^−^ ion vs time quantity for three homogeneous reaction rate constants.

The quantity of other ions (Fe^2+^, OH^–^) decreases, as seen in Figure 15 and Figure 16. However, the overall result is dominated by Cl^−^ ions. On the other hand, the initial fall of conductivity is related to the migration of ions from capillary pores to the cementitious matrix (due to the fact that at the beginning, concentrations of ions are higher in capillary pores) where mobilities are by three orders of magnitude smaller. For the dependence of conductivity (Figure 14) on the rate constant *k* the following trend is observed: the higher its value, the smaller the conductivity, since the consumption of Fe^2+^ and OH^–^—leading to the overall fall of ionic concentration—is faster for higher *k* values.

Figure 16 shows the total quantity of hydroxide ions vs. time and its dependence on the reaction rate (10). The case k=0 means no reaction at all. As expected, for a larger rate value, there is a larger fall of OH^–^ due to the quicker consumption of OH^−^ ions.

### 6.2. The Influence of Oxygen Diffusion Coefficient

**Figure 17:**
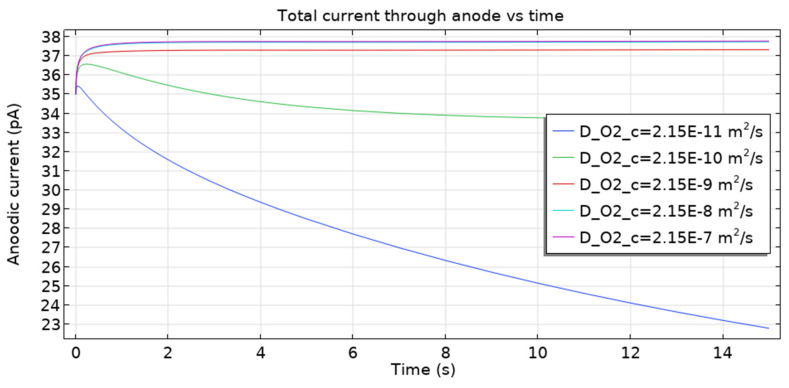
The total current through the cathode is a function of time for various values of oxygen diffusivity.

The oxygen diffusivity in concrete can change depending on the degree of water saturation [66]. Figure 17 presents the dependence of the total anodic current (hence the corrosion rate) on oxygen diffusivity. We conclude that in each case, the corrosion rate approaches a constant value (steady-state) and behaves monotonically for three values (*D*_O2_ = 2.15 · 10^−7^, 2.15 · 10^−8^, 2.15 · 10^−9^ m^2^ s^−1^). However, the anodic current density goes through a maximum (about 36.5 pA and 3.54 pA) followed by a monotonic fall for *D*_O2_ = 2.15 · 10^−10^, 2.15 · 10^−10^ m^2^·s^−1^.

**Figure 18:**
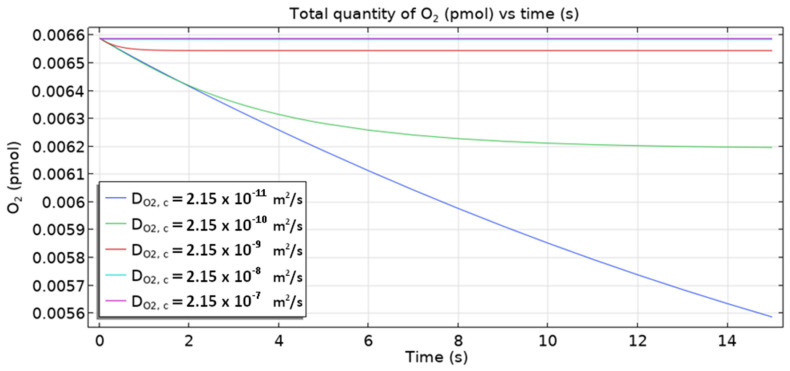
The total quantity of oxygen in the sample as a function of time for different values of oxygen diffusivity.

Figure 18 shows the total quantity of oxygen in the sample vs. times for five values of the oxygen diffusion coefficient (2.5·10^−11^–2.5·10^−7^ m^2^ s^−1^). A decrease in total quantity is always introduced, and the smaller the diffusivity value, the larger this decrease is. Oxygen is consumed on the cathode part of the rebar. However, its ingress from the top part of the sample is much smaller than its depletion in the sample and on the rebar cathodic site, hence the fall in overall quantity. For smaller diffusivities, less oxygen is transported—hence the observed dependence on the diffusivity.

**Figure 19:**
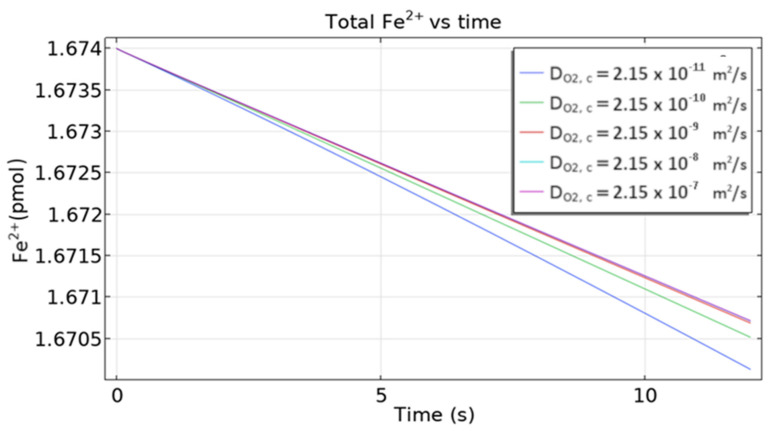
The total quantity of Fe^2+^ ion vs time for different oxygen diffusion coefficients.

Figure 19 shows a change in time of the total quantity of ferrous ions in the sample for several oxygen diffusivities. Generally, for smaller diffusivity, a bigger fall of the curve is observed. This phenomenon is not easy to rationalize as several factors contribute to the quantity of Fe^2+^.

### 6.3. The Influence of Chloride Concentration

**Figure 20:**
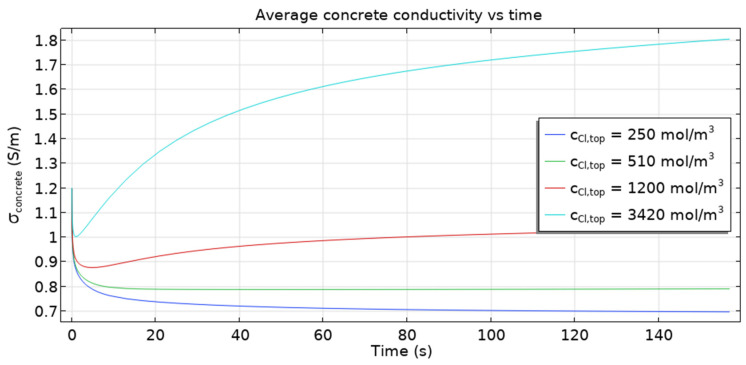
Average conductivity in the sample as a function of time for different boundary concentrations of chloride ions, *c*_Cl,top_. As expected, for bigger concentrations, the conductivities are higher.

Figure 20 shows that the time−dependent average conductivity depends on the boundary concentration of chloride ions. Interestingly, qualitative behavior differs for $c_{{\mathrm{Cl}}^{-},top}\in \{250,\;510\}$ and $c_{{\mathrm{Cl}}^{-},top}\in \{1200,\;3420\}$ (mol/m^3^). In the first case (lower $c_{{\mathrm{Cl}}^{-},top})$ curves are monotonically decreasing, while in the second case (higher $c_{{\mathrm{Cl}}^{-},top})$ the behavior is not monotonic. The initial sharp drop is caused by the ingress of chlorides from the capillary into cementitious matrix where diffusivities are much smaller, but eventually, the ingress of large quantities of Cl^–^ ions through the boundary prevails (provided that some threshold of boundary concentration is surpassed; here it is around 1200 mol/m^3^).

**Figure 21:**
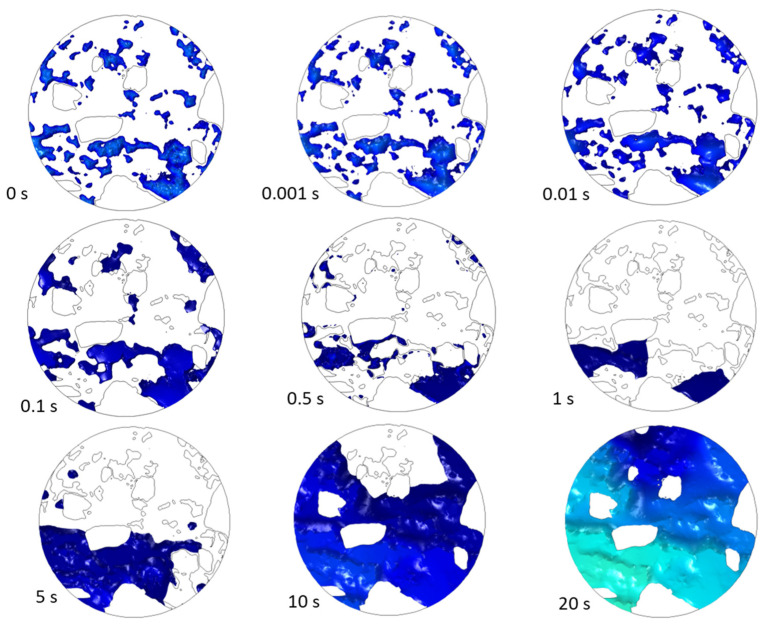
Regions on the rebar surface where the chloride threshold was exceeded as they evolve in time.

The ingress of chlorides into concrete can initiate reinforcement corrosion by destroying a protective layer (amorphic mixture of hydrated iron oxides/hydroxides) by a process known as depassivation. It can ultimately result in the breakdown of the reinforced concrete structure. An essential parameter for identifying possible locations and times of depassivation is the so-called chloride threshold value. It is defined as the minimum concentration of chloride on the surface of a rebar that can commence the depassivation of the steel. Our model can provide these regions by showing how the concentration on the rebar surface is developing spatially and temporally. Figure 21 displays exemplary maps of chloride concentration, which is above some threshold value on the rebar surface as they evolve in time. Such maps can help identify the regions in the structure most susceptible to chloride attack and failure due to reinforcing rod corrosion.

Examples in Section 6 demonstrate some of the possibilities and advantages which may result from computational analysis based on a 3D multi-ion corrosion model. The presented model can be utilized for more detailed investigation and understanding of reinforcement corrosion and opens new possibilities in more accurately predicting concrete durability. The model’s accuracy depends on the accuracy of model parameters, particularly parameters describing boundary conditions (Butler-Volmer equations). The electrochemical properties of steel in reinforced concrete vary significantly in the literature [68]. Most recent results [69] show the influence of chloride concentration on the electrochemical parameters of active steel. Boundary conditions considering dynamic changes of electrochemical parameters of steel in concrete can be easily implemented in this model because the transport of chloride ions is calculated in time and space domains.

## 7. 3D vs. 2D Corrosion Model

**Figure 22:**
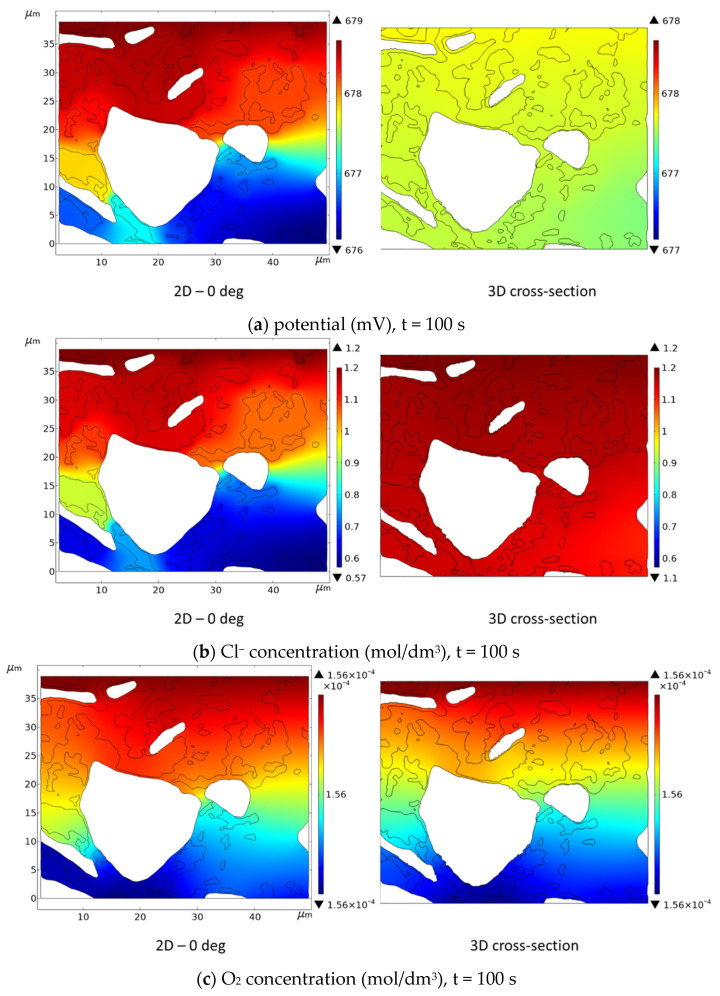

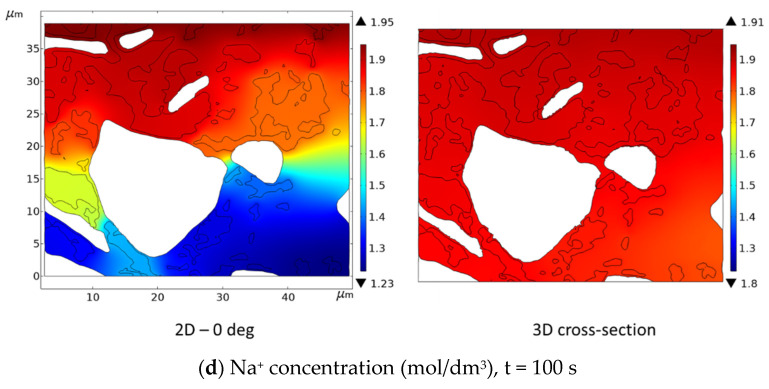
Comparison of electric potential (**a**) and selected concentration of species (**b**–**d**) obtained from direct 2D calculations and from cross-section (0 deg—see Figure 4) of 3D calculations.

In this section, we compare results for 2D and 3D models of steel reinforcement corrosion for a real cementitious material microstructure that was determined using nano-XCT. 2D calculations were performed for six virtual cross-sections (see Figure 4). Figure 22 shows the results for a selected cross-section at 0 deg.

Although the electric potential in both cases (2D and 3D) is approximately in the same range, it differs in its distribution. It is flatter in the 3D case, see Figure 22a. The transport of chloride ions is faster for the 3D geometry, and its concentration near the cathode and anode reaches higher values for the 3D geometry (~1.1 mol/dm^3^) than for the 2D case (~0.57 mol/dm^3^)—see Figure 22b. On the other hand, the oxygen concentrations in both cases are very similar (Figure 22c). At the same time, there is a noticeable difference for sodium ions both in the distribution (3D cases giving more flat concentration) and in the ranges, as seen in Figure 22d.

**Figure 23:**
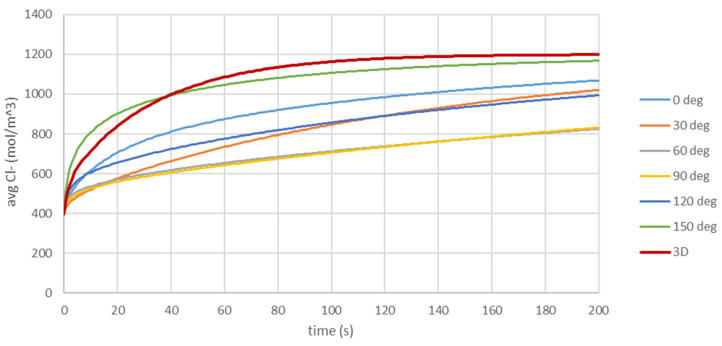
Comparison of the average chloride ion concentration in the sample as a function of time obtained from 3D model (red line) and 2D models (cross-section 0 deg, …, 150 deg).

**Figure 24:**
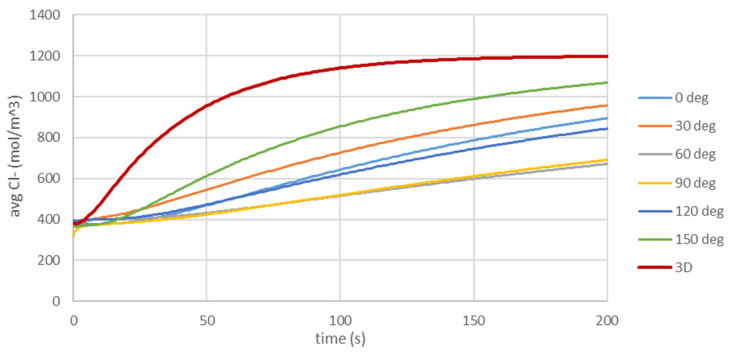
Comparison of the average chloride ion concentration at the surface of the rod as a function of time obtained from 3D model (red line) and 2D models (cross-section 0 deg, …, 150 deg).

Figure 23 compares the average chloride ion concentration in the sample as a function of time calculated for 3D and 2D models (for different cross-sections). In most cases, the calculated average chloride ion concentration in 2D models underestimates the chloride ion concentration. The relative difference between 3D and 2D models most of the time is ~30%. This effect is even stronger at the surface of the rod (Figure 24), where the calculated average chloride ion concentration in the 2D model can differ from that in the 3D model by even ~45%. Such big differences in chloride ion concentrations will significantly influence the corrosion process.

The differences between 2D and 3D calculations may be affected by the microstructure of the cementitious material and the overall geometry of a specimen. Thus, in our opinion, in the case of the real material’s geometry, reliable results may require calculations in 3D geometry.

## 8. Summary and Conclusions

This paper has developed and applied a comprehensive approach to describing corrosion processes in steel-reinforced concrete. The method is based on obtaining the microstructure of the cementitious material from nano-XCT and subsequently processing the data to generate a suitable mesh for FEM computations. Finally, a general set of PDEs is applied. Boundary conditions consider a dynamic transport of all relevant species (ionic and neutral) and the charge transfer kinetics at rebars anodic and cathodic sites to solve for concentrations, electric potential, and current distribution. Others can be derived from these quantities, for example, the loss of rebar mass or electrical conductivity. There are substantial differences between the predictions of simplified models and the 3D multi-ion corrosion model proposed in the paper. The comparison of the present study with a simplified state-of-the-art model (Ožbolt) shows the advantage of the model proposed in this paper. Particularly important is the prediction of the loss of rebar mass which may be larger than predicted in a simplified model.

In a real corroding system of reinforced concrete several species are present. To obtain a reliable simulation of the degradation process, these species have to be included in the modelling. In particular, the movement of ions influences the spatial distribution of electrical potential and electrical conductivity over time and indirectly on the corrosion rate. In simplified descriptions, like the Ožbolt model, the spatial distributions of potential practically do not change with time. This model considers only the diffusion of oxygen atoms, which slightly changes its concentration near the cathode where the concentration overpotential may slightly change the potential values near the boundary. However, there is no time dependence on the potential inside the sample based on the Laplace equation.

We have also considered the kinetics of iron dissolution on anodic sites more accurately. The two reactions (anodic and cathodic) are somewhat independent (of course, with global constrain of total charge balance).

The image-based multiscale model proposed in this paper considers the real 3D microstructure data of concrete as determined experimentally using XCT as a basis for distinguishing between capillary pores and gel pores instead of treating concrete as homogeneous medium with only bulk parameters averaging intricate internal morphology. Incorporating the microstructure of the cementitious material into the model gives deeper insight into the local factors influencing the corrosion, such as electrical potential and current distribution, chloride ion content etc. For example, the 3D microstructure of concrete allows for describing localized corrosion. It was shown that concentrations of ions and oxygen and the electric potential have a substantial influence on the corrosion reactions at the surface of the steel reinforcement. The concrete’s morphology and non-homogeneous chloride ion concentration on the rod surface can cause pitting corrosion. The electric potential via Butler–Volmer boundary conditions has a decisive impact on the corrosion rate and, consequently, on the corrosion kinetics of the steel reinforcement.

Since the 3D microstructure significantly impacts the corrosion process of rebars in concrete (which comprise both interfacial and bulk phenomena), simple 1D or 2D models are not applicable for describing the corrosion process in real cementitious materials.

In this paper, we showed that the real 3D microstructure of a concrete sample obtained from XCT, combined with a multi-species system of transport and electric potential equations, enables a more realistic description of ion migration and reactions in the bulk concrete and on the rebar surface, and in consequence, a better evaluation of anodic and cathodic currents which are ultimately responsible for the loss of reinforcement mass and its location. 

In this model, we have consciously introduced a few simplifications, such as neglecting chloride binding and thermodynamic ion activities and their influence on the transport of ions in cementitious materials. Although they have already been investigated individually [67,70], their incorporation into the proposed model will be the subject of further development. The article aimed to show the potential of the proposed model, which considers based 3D materials microstructure, materials transport, and reactions of many species in cementitious materials. The presented results are in the micro- and nano-scale. The extension of the model to the meso- and macro-scale needs further investigation. For this extension, the representative element volume (REV) [71] should be determined, and a homogenization [72] might be applied. Moreover, the model at the mesoscale level might be compared with the results of LIBS analysis [73].

## Figures and Tables

**Table 1:** Nano-XCT application for cementitious materials—comparison of recent works.

Author(s)	Method/Apparatus	What Was Observed	ROI (One Dimension)	Resolution
Brisard et al. [21]	Transmission X-ray microscopy	Portland cement paste	2 µm	30 nm
Jackson et al. [22]	HZB-TXM	Al-tobermorite crystal clusters	5 µm	50 nm
Cuesta et al. [23]	Ptychographic XCT	ye’elimite, ye’elimite- gypsum and CSA paste	50 µm	40.8 nm
da Silva et al. [24]	Ptychographic XCT	alite-based hardened cement paste	40 µm	43.7 nm
Hu et al. [25]	Hard X-ray Nanoprobe	Ca_3_SiO_5_ powder	20 µm	15.6 nm
Provis et al. [26]	Hard X-ray Nanoprobe	Fly ash	10 µm	30 nm
Shirani et al. [27]	Ptychographic XCT	CA pastes hydrated for 5 months	15 µm	39.84 nm
Trtik et al. [28]	Ptychographic XCT	hardened cement paste	31.4 µm	43.6 nm
Bullard et al. [30]	Hard X-ray Nanoprobe	Ca_3_SiO_5_ powder	20 µm	15.6 nm
Chae et al. [31]	Review			
Chen et al. [32]	Transmission X-ray microscopy	Hydrated C_3_S paste	50 µm	50 nm

**Table 2:** Properties of the mortar used for the measurements.

Bending Strength//Compression Strength (MPa)			Absorption (%)		Test Results from Mercury Porosimetry		
Maturation Time			Maturation Time		Porosity (%)	Cumulative Pore Volume (mm^3^/g)	Density (kg/m^3^)
2 Days	28 Days	90 Days	28 Days	90 Days			
6.2//38.9	7.5//48.4	7.6//54.1	8.0	9.0	16.8	79.1	2130

**Table 3:** Physicochemical parameters used in simulations.

Parameters	Value
$\mathrm{Anodic}\;\mathrm{exchange}\;\mathrm{current}\;\mathrm{density},\;i_{Fe}\;(A/m^{2})$	1.875 × 10^−4^
$\mathrm{Cathodic}\;\mathrm{exchange}\;\mathrm{current}\;\mathrm{density},\;i_{O_{2}}\;(A/m^{2})$	6.250 × 10^−6^
$\mathrm{Anodic}\;\mathrm{equilibrium}\;\mathrm{potential},\;E_{eq,Fe}\;(V\;\mathrm{vs}.\;\mathrm{SCE})$	−0.780
$\mathrm{Cathodic}\;\mathrm{equilibrium}\;\mathrm{potential},\;E_{eq,O_{2}}\;(V\;\mathrm{vs}.\;\mathrm{SCE})$	0.160
$\mathrm{Tafel}\;\mathrm{slope}\;\mathrm{for}\;\mathrm{anodic}\;\mathrm{reaction},\;\beta_{Fe}\;(V/\mathrm{dec})$	0.06
$\mathrm{Tafel}\;\mathrm{slope}\;\mathrm{for}\;\mathrm{cathodic}\;\mathrm{reaction},\;\beta_{O_{2}}\;(V/\mathrm{dec})$	0.16
$\mathrm{Oxygen}\;\mathrm{diffusivity},\;D_{O_{2}}\;(m^{2}/s)$	2.15 × 10^−9^
$\mathrm{Sodium}\;\mathrm{anion}\;\mathrm{diffusivity},\;D_{{\mathrm{Na}}^{+}}\;(m^{2}/s)$	1.33 × 10^−12^
$\mathrm{Iron}(\mathrm{II})\;\mathrm{cation}\;\mathrm{diffusivity},\;D_{{\mathrm{Fe}}^{2+}}\;(m^{2}/s)$	1.05 × 10^−12^
$\mathrm{Chloride}\;\mathrm{anion}\;\mathrm{diffusivity},\;D_{{\mathrm{Cl}}^{-}}\;(m^{2}/s)$	2.01 × 10^−12^
$\mathrm{Hydroxide}\;\mathrm{anion}\;\mathrm{diffusivity},\;D_{{\mathrm{OH}}^{-}}\;(m^{2}/s)$	5.29 × 10^−12^
Homogeneous rate constant, *k* (m^3^/(mol·s^3^))	3.5 × 10^−3^

## Data Availability

Data is contained within the article.

## Appendix A. Reformulation of the Multi-Component Corrosion Problem

The diffusion-migration problem defined by (1), (2), electroneutrality (6), and flux-source boundary (7) conditions can, in theory, be solved directly without any prior rearrangements. However, this pristine form forms a partial differential algebraic system of equations (PDAEs) that might pose some difficulties. Thus, it is advantageous to reformulate the problem (equations and boundary conditions) so that the electroneutrality condition is somehow embedded into equations. Let us divide each Equation (1) by *D_i_* and sum over i=1,…,N:

(A1) $$\frac{\partial }{\partial t}\left(\phi \sum_{i=1}^{N}\frac{c_{i}}{D_{i}^{eff}}\right)\;+\;\mathrm{div}\;\left(\sum_{i=1}^{N}\frac{1}{D_{i}^{eff}}J_{i}\right)\;=\;\sum_{i=1}^{N}\frac{1}{D_{i}^{eff}}R_{i}.$$

Using flux (2), we have $\sum_{i=1}^{N}\frac{1}{D_{i}^{eff}}J_{i}=\sum_{i=1}^{N}\frac{1}{D_{i}^{eff}}(-D_{i}^{eff}\nabla c_{i}+B_{i}^{eff}c_{i}E)\overset{B_{i}=\frac{z_{i}F}{RT}D_{i}}{=}-\nabla \left(\sum_{i=1}^{N}c_{i}\right)+\frac{F}{RT}\left(\sum_{i=1}^{N}z_{i}c_{i}\right)E$ which, after applying the electroneutrality condition, turns (A1) into

(A2) $$\frac{\partial }{\partial t}\left(\phi \sum_{i=1}^{N}\frac{c_{i}}{D_{i}^{eff}}\right)\;+\;\mathrm{div}\;\nabla \left(-\sum_{i=1}^{N}c_{i}\right)\;=\sum_{i=1}^{N}\frac{1}{D_{i}^{eff}}R_{i}.$$

We will eliminate one selected ionic component from the system. Let this chosen component has the index $i_{0}\in \{1,\ldots ,N\}.$ Multiply Equation (A2) by $z_{i_{0}}$ and invoking the electroneutrality condition again

$$\begin{array}{l} \frac{\partial }{\partial t}\left(\phi \sum_{i=1}^{N}\frac{z_{i_{0}}c_{i}}{D_{i}}\right)\;+\;\mathrm{div}\;\nabla \left(-\sum_{i=1}^{N}z_{i_{0}}c_{i}\right)\;=\;\sum_{i=1}^{N}\frac{z_{i_{0}}}{D_{i}}R_{i},\;\\ \frac{\partial }{\partial t}\left(\phi \frac{z_{i_{0}}c_{i_{0}}}{D_{i_{0}}}+\phi \sum_{i\neq i_{0}}\frac{z_{i_{0}}c_{i}}{D_{i}^{eff}}\right)\;+\;\mathrm{div}\;\nabla \left(-z_{i_{0}}c_{i_{0}}-\sum_{i\neq i_{0}}z_{i_{0}}c_{i}\right)\;=\;\sum_{i=1}^{N}\frac{z_{i_{0}}}{D_{i}^{eff}}R_{i},\\ z_{i_{0}}c_{i_{0}}=-\sum_{i\neq i_{0}}z_{i}c_{i}\;\Rightarrow \;\frac{\partial }{\partial t}\left(-\phi \sum_{i\neq i_{0}}\frac{z_{i}c_{i}}{D_{i_{0}}^{eff}}+\phi \sum_{i\neq i_{0}}\frac{z_{i_{0}}c_{i}}{D_{i}}\right)\;+\;\mathrm{div}\nabla \left(\sum_{i\neq i_{0}}z_{i}c_{i}-\sum_{i\neq i_{0}}z_{i_{0}}c_{i}\right)\;=\;\sum_{i=1}^{N}\frac{z_{i_{0}}}{D_{i}^{eff}}R_{i},\\ \frac{\partial }{\partial t}\phi \sum_{i\neq i_{0}}\left(\frac{z_{i_{0}}}{D_{i}^{eff}}-\frac{z_{i}}{D_{i_{0}}^{eff}}\right)c_{i}\;+\;\mathrm{div}\;\nabla \left(\sum_{i\neq i_{0}}(z_{i}-z_{i_{0}})c_{i}\right)\;=\;\sum_{i=1}^{N}\frac{z_{i_{0}}}{D_{i}^{eff}}R_{i}. \end{array}$$

Multiply now the last equality by $-D_{i_{0}}^{eff}$ and rearrange to get

(A3) $$\sum_{i\neq i_{0}}\phi \left(z_{i}-z_{i_{0}}\frac{D_{i_{0}}^{eff}}{D_{i}^{eff}}\right)\frac{\partial c_{i}}{\partial t}\;+\;\mathrm{div}\left(-D_{i_{0}}^{eff}\sum_{i\neq i_{0}}(z_{i}-z_{i_{0}})\nabla c_{i}\right)\;=-\sum_{i=1}^{N}z_{i_{0}}\frac{D_{i_{0}}^{eff}}{D_{i}^{eff}}R_{i},$$

where the summing is over all ionic species {1,…,N} except $i_{0}:\;\sum_{i\neq i_{0}}=\sum_{i=1,i\neq i_{0}}^{N}.$ Notice that the Equation (A3) has the form of the mass balance law with the “flux” and “reaction” terms:

(A4) $$J_{i_{0},E}\;=\;-D_{i_{0}}^{eff}\sum_{i\neq i_{0}}(z_{i}-z_{i_{0}})\nabla c_{i},\;R_{i_{0},E}=-\sum_{i=1}^{N}z_{i_{0}}\frac{D_{i_{0}}^{eff}}{D_{i}^{eff}}R_{i}.$$

We have to reformulate the boundary condition for the component $i_{0}$ according to the form of Equation (A3):

$$\begin{array}{l} -n\cdot J_{i}=g_{i}\;\Rightarrow \;-n\cdot \left(\sum_{i=1}^{N}\frac{1}{D_{i}^{eff}}J_{i}\right)=\sum_{i=1}^{N}\frac{g_{i}}{D_{i}^{eff}}\;\overset{el.\;neutral.\;cond.}{\Rightarrow }\;-n\cdot \left(\sum_{i=1}^{N}\left(-\nabla c_{i}\right)\right)=\sum_{i=1}^{N}\frac{g_{i}}{D_{i}^{eff}}\;|\cdot \;z_{i_{0}}\\ -n\cdot \left(\nabla (-z_{i_{0}}c_{i_{0}}-\sum_{i\neq i_{0}}z_{i_{0}}c_{i})\right)=\sum_{i=1}^{N}\frac{z_{i_{0}}g_{i}}{D_{i}^{eff}},\;\mathrm{but}\;z_{i_{0}}c_{i_{0}}=-\sum_{i\neq i_{0}}z_{i}c_{i},\;\mathrm{hence}\\ \left.-n\cdot \left(\nabla (\sum_{i\neq i_{0}}z_{i}c_{i}-\sum_{i\neq i_{0}}z_{i_{0}}c_{i})\right)=\sum_{i=1}^{N}\frac{z_{i_{0}}g_{i}}{D_{i}^{eff}}\;\right|\;\cdot \;-D_{i_{0}}^{eff}\\ -n\cdot \left(-D_{i_{0}}^{eff}\sum_{i\neq i_{0}}(z_{i}-z_{i_{0}})\nabla c_{i}\right)=-z_{i_{0}}D_{i_{0}}^{eff}\sum_{i=1}^{N}\frac{g_{i}}{D_{i}^{eff}}, \end{array}$$

what gives the following boundary condition

(A5) $$-n\cdot J_{i_{0},E}=-z_{i_{0}}D_{i_{0}}^{eff}\sum_{i=1}^{N}\frac{g_{i}}{D_{i_{0}}^{eff}}\;\mathrm{on}\;\partial \Omega \;.$$

In summary, the PDE system with embedded electroneutrality condition and electric field E=−∇φ expressed by electric potential *φ* is as follows (in Ω):

(A6) $$\left\{\begin{array}{l} \phi \frac{\partial c_{i}}{\partial t}\;+\;\mathrm{div}\;J_{i}\;=\;R_{i},\;J_{i}=-D_{i}^{eff}\nabla c_{i}-D_{i}^{eff}z_{i}\frac{F}{RT}c_{i}\nabla \varphi ,\;(i=1,\ldots N+N_{0},\;i\neq i_{0}),\\ \sum_{i\neq i_{0}}\phi \left(z_{i}-z_{i_{0}}\frac{D_{i_{0}}^{eff}}{D_{i}^{eff}}\right)\frac{\partial c_{i}}{\partial t}\;+\;\mathrm{div}\;J_{i_{0},E}\;=\;R_{i_{0},E},\;(i=i_{0}),\\ J_{i_{0},E}\;=\;-D_{i_{0}}^{eff}\sum_{i\neq i_{0}}(z_{i}-z_{i_{0}})\nabla c_{i},\;R_{i_{0},E}=-\sum_{i=1}^{N}z_{i_{0}}\frac{D_{i_{0}}^{eff}}{D_{i}^{eff}}R_{i}. \end{array}\right.$$

with boundary conditions (on ∂Ω):

(A7) $$\left\{\begin{array}{l} -n\cdot J_{i}=g_{i}\;i=1,\ldots ,N+N_{0},\;i\neq i_{0},\\ -n\cdot J_{i_{0},E}=-\sum_{i=1}^{N}z_{i_{0}}\frac{D_{i_{0}}^{eff}}{D_{i}^{eff}}g_{i}\;i\neq i_{0}. \end{array}\right.$$
